# Rescuing Dicer expression in inflamed colon tissues alleviates colitis and prevents colitis-associated tumorigenesis

**DOI:** 10.7150/thno.41894

**Published:** 2020-04-27

**Authors:** Xiaoli Wu, Xiao Chen, Hui Liu, Zhong-Wei He, Zheng Wang, Lin-Jie Wei, Wei-Yun Wang, Shouhui Zhong, Qin He, Zhechao Zhang, Rongying Ou, Jian Gao, Youchun Lei, Wenjun Yang, Guanbin Song, Yi Jin, Lingli Zhou, Yunsheng Xu, Kai-Fu Tang

**Affiliations:** 1 Department of Gastroenterology, The First Affiliated Hospital of Wenzhou Medical University, Wenzhou 325015, Zhejiang, P.R. China; 2 Digestive Cancer Center, The First Affiliated Hospital of Wenzhou Medical University, Wenzhou 325015, Zhejiang, P.R. China; 3 Key Laboratory of Diagnosis and Treatment of Severe Hepato-Pancreatic Diseases of Zhejiang Province, The First Affiliated Hospital of Wenzhou Medical University, Wenzhou 325015, Zhejiang, P.R. China; 4 Department of Dermato-Venereology, The First Affiliated Hospital of Wenzhou Medical University, Wenzhou 325015, Zhejiang, P.R. China; 5 Department of Gynecology, The First Affiliated Hospital of Wenzhou Medical University, Wenzhou 325015, Zhejiang, P.R. China; 6 Department of Gastroenterology, The Second Affiliated Hospital of Chongqing Medical University, Chongqing 400010, P.R. China; 7 Department of Gastroenterology, The Sixth People's Hospital of Chongqing, Chongqing 404100, P.R. China; 8 Department of Hepatobiliary Surgery, The First Affiliated Hospital of Wenzhou Medical University, Wenzhou 325015, P.R. China; 9 Department of Bioengineering, College of Bioengineering, Chongqing University, Key Laboratory of Biorheological Science and Technology, Ministry of Education, Chongqing 400030, P.R. China; 10 Department of Pathology, The First Affiliated Hospital of Wenzhou Medical University, Wenzhou 325000, Zhejiang, P.R. China; 11 Department of Pathology, The Second Affiliated Hospital of Wenzhou Medical University, Wenzhou 325015, Zhejiang, P.R. China

**Keywords:** Dicer, colitis-associated carcinogenesis, anastrozole, berberine, pranoprofen

## Abstract

Chronic inflammation is known to promote carcinogenesis; Dicer heterozygous mice are more likely to develop colitis-associated tumors. This study investigates whether Dicer is downregulated in inflamed colon tissues before malignancy occurs and whether increasing Dicer expression in inflamed colon tissues can alleviate colitis and prevent colitis-associated tumorigenesis.

**Methods**: Gene expression in colon tissues was analyzed by immunohistochemistry, immunoblots, and real-time RT-PCR. Hydrogen peroxide or N-acetyl-L-cysteine was used to induce or alleviate oxidative stress, respectively. Mice were given azoxymethane followed by dextran sulfate sodium to induce colitis and colon tumors. Berberine, anastrozole, or pranoprofen was used to rescue Dicer expression in inflammatory colon tissues.

**Results**: Oxidative stress repressed Dicer expression in inflamed colon tissues by inducing miR-215 expression. Decreased Dicer expression increased DNA damage and cytosolic DNA and promoted interleukin-6 expression upon hydrogen peroxide treatment. Dicer overexpression in inflamed colon tissues alleviated inflammation and repressed colitis-associated carcinogenesis. Furthermore, we found that anastrozole, berberine, and pranoprofen could promote Dicer expression and protect cells from hydrogen peroxide-induced DNA damage, thereby reducing cytosolic DNA and partially repressing interleukin-6 expression upon hydrogen peroxide treatment. Rescuing Dicer expression using anastrozole, berberine, or pranoprofen in inflamed colon tissues alleviated colitis and prevented colitis-associated tumorigenesis.

**Conclusions**: Dicer was downregulated in inflamed colon tissues before malignancy occurred. Decreased Dicer expression further exaggerated inflammation, which may promote carcinogenesis. Anastrozole, berberine, and pranoprofen alleviated colitis and colitis-associated tumorigenesis by promoting Dicer expression. Our study provides insight into potential colitis treatment and colitis-associated colon cancer prevention strategies.

## Introduction

Dicer is a key component of the RNA interference pathway and is essential for the biogenesis of microRNAs (miRNAs) and small interfering RNAs (siRNAs) 1. Recent evidence indicates that Dicer is frequently downregulated in tumor tissues, and that decreased Dicer expression promotes carcinogenesis 1, 2. Somatic mutations in Dicer have been identified in various cancer types 1, 3, 4, and heterozygous germline Dicer mutations have been shown to increase the risk of a variety of tumors, particularly in the lungs, kidneys, ovaries, and thyroid 1, 5. Although Dicer has been proposed to function as a haploinsufficient tumor suppressor 3, other studies have indicated that the full loss of Dicer does not preclude carcinogenesis 6, 7.

Global control of protein synthesis is crucial for cancer development and progression, as highly proliferating cancer cells require increased protein synthesis 8. Decreased Dicer expression may promote protein synthesis through several mechanisms. First, Dicer is essential for miRNA biogenesis, and miRNAs repress protein synthesis by inhibiting translation or degrading their target mRNAs 1; therefore, decreased Dicer expression may lead to increased protein synthesis due to globally impaired miRNA expression 2, 9, 10. Second, Dicer can process full-length tRNA into small fragments called tRNA-derived small RNA (tsRNA) that inhibit protein translation 11, 12, therefore, decreased Dicer expression may lead to enhanced protein synthesis due to increased full-length tRNA levels and decreased tsRNA levels. Lastly, Dicer can process 7SL RNA into small fragments, which impair signal recognition particle (SRP) formation and thereby inhibit SRP-mediated protein targeting 13, 14. SRP couples the synthesis of nascent proteins to their correct cellular destinations 15; therefore, decreased Dicer expression may promote localization of nascent proteins to their correct cellular destinations. Altogether, these findings indicate that decreased Dicer expression may promote carcinogenesis by increasing protein synthesis.

Dicer is essential for DNA repair, and decreased Dicer expression has been shown to reduce the efficiency of DNA repair and leads to accumulation of DNA damage 16-21. DNA damage can promote inflammation by inducing the expression of NKG2D ligands; upregulation of NKG2D ligands can initiate immune-mediated tissue damage and induce inflammation 22, 23. Moreover, DNA damage leads to the accumulation of DNA and micronuclei in the cytoplasm; this cytosolic DNA triggers the production of proinflammatory cytokines such as type I interferons and interleukin-6 (IL-6) 24, 25. Inflammation is indispensable in neoplastic processes and promotes cancer initiation and the subsequent proliferation, survival, and migration of cancer cells 26. Knockout of Dicer in intestinal epithelial cells was found to induce inflammation, and Dicer heterozygous mice were more likely to develop colitis-associated tumors 27. Therefore, in addition to its mutational consequences, decreased Dicer expression may promote tumorigenesis by inducing inflammation.

It has been reported that Dicer mRNA levels are slightly decreased in colon cancer tissues but significantly increased in rectum cancer tissues compared with normal mucosa tissues 28. Moreover, Dicer mRNA levels in stage I and stage II colorectal tumor tissues are lower than in stage III and stage IV tumor tissues 29. Inflammation plays pivotal roles in the development of colitis-associated colon cancer 26, 30. However, the dynamic pattern of Dicer expression during colitis-associated carcinogenesis remains unclear. Accordingly, in the current study, we examined whether Dicer is downregulated in inflamed colon tissues before a malignant change occurs. In addition, we investigated whether rescue of Dicer expression in inflamed colon tissues alleviates colitis and colitis-associated tumorigenesis.

## Methods

### Cell culture and drug treatment

FHC, CCD-18Co, and THP-1 cell lines were purchased from the American Type Culture Collection (Manassas, VA) and HEK293T was obtained from the Cell Bank of the Chinese Academy of Sciences (Shanghai, China). CCD-18Co and HEK293T were grown in DMEM (Hyclone, Logan, UT) supplemented with 10% FBS, while FHC was grown in RPMI 1640 (Hyclone) supplemented with 10% FBS. THP-1 cells were cultured in RPMI 1640 containing 10% FBS and 50 μM β-mercaptoethanol. Cells were cultured at 37 °C in a 5% CO_2_ humidified incubator. All cell lines were mycoplasma free, and cells passaged in our laboratory > 6 months after receipt were authenticated by genetic profiling using polymorphic short tandem repeat loci. To identify drugs that can promote Dicer expression, we screened a drug library (Targetmol, Wellesley Hills, MA), which contains 1800 US-FDA approved drugs. Briefly, cells were seeded in 6-well plates to reach 80% confluence, and then treated with different drugs at a concentration of 30 μM. Dicer protein levels were determined using western blotting 24 h after treatment.

### Human colon tissue specimens

In total, 91 normal colon tissue samples and 102 inflammatory bowel diseases (IBD) colon tissue samples were collected from patients who underwent enteroscopy and biopsy at the First Affiliated Hospital of Wenzhou Medical University (Wenzhou, China). Informed consent was obtained from all patients for the collection and use of clinical samples, and the study was approved by the Scientific Ethics Committee of the First Affiliated Hospital of Wenzhou Medical University. Samples were either rapidly frozen using liquid nitrogen or fixed in formalin solution. Rapidly frozen samples were stored at -80 °C to further extract RNA and protein for miR-215 and Dicer quantification.

### Mouse studies

Acute colitis was induced in six-week-old male mice by oral administration of 3% dextran sulfate sodium (DSS; MP Biomedicals, Santa Ana, CA) in drinking water for 7 days, followed by 2 days of normal drinking water. All mice were euthanized on day 9.

To induce colitis-associated colon cancers, six-week-old male C57BL/6 mice were injected intraperitoneally with 12.5 mg/kg azoxymethane (AOM; Sigma-Aldrich, St. Louis, MO). After 5 days, chronic colitis was induced by 3 cycles of DSS. One cycle of DSS was defined as 5 days of DSS administration followed by a recovery period of 16 days on normal drinking water. All mice were euthanized on day 92. Control mice received normal drinking water throughout the duration of the experiment.

To introduce adenoviruses or lentiviruses to mouse colon tissues, mice were anesthetized and given an intrarectal enema of 100 μL 50% ethanol. Three hours after the enema, 100 μL lentivirus solution containing 10^8^ titers lentivirus or 10^9^ adenovirus was intrarectally instilled from the mouse anus. The mice were inverted for 30 s after intrarectal administration ofvirus to prevent leakage. The mouse Dicer overexpression adenovirus or control adenovirus was customized from Cyagen Biosciences Inc. (Guangzhou, China). Mouse Dicer shRNA or control lentivirus was purchased from Origene (Rockville, MD).

To relieve oxidative stress in inflamed tissues, NAC (Sigma-Aldrich) was added to drinking water at a final concentration of 2%.

All mice were reared and handled in accordance with the Institutional Guidelines on Animal Usage and Maintenance of Wenzhou Medical University.

### Plasmids, siRNAs, miRNAs, and transfection

Cells were transfected with siRNAs, miRNAs, or plasmids using Lipofectamine 2000 (Life Technologies, Carlsbad, CA) according to the manufacturer's instructions. miRNA mimics, the miR-215 inhibitor (anti-miR-215), and the respective negative controls (miR-Con and anti-miR-Con) were purchased from RiboBio (Guangzhou, China). Control and Dicer siRNAs were obtained from Life Technologies and the sequences were described previously 16. The human Dicer overexpression plasmid pDESTmycDICER (pDicer) was obtained from Addgene (Cambridge, MA).

### Western blotting

The total cell lysate was subjected to SDS-PAGE and transferred to a polyvinylidene fluoride membrane (Millipore). Blots were incubated with primary antibodies, followed by incubation with horseradish peroxidase (HRP)-conjugated secondary antibodies and detection with ECL plus reagents (GE Healthcare, Chicago, IL). Primary antibodies used were anti-Dicer (ab14601; Abcam, Cambridge, UK), anti-γ-Tubulin (BM1606; BosterBio, Wuhan, China), and anti-GAPDH (2188; Cell Signaling Technology, Danvers, MA). Relative Dicer expression levels were calculated via densitometry and normalized to GAPDH expression using Image J software (http://rsb.info.nih.gov/ij/).

### Cell proliferation assay

Cell proliferation was assessed using the CellTiter 96 Aqueous One Solution Cell Proliferation Assay (MTS) kit (Promega, Madison, WI) according to the manufacturer's instructions.

### Immunohistochemistry, hematoxylin and eosin (HE) staining, and TUNEL assay

Immunohistochemistry was performed as described previously 18. To assay inflammatory cell infiltration, the hydrated tissue sections were stained with HE, dehydrated in ethanol, cleared in xylene, and mounted on coverslips. Inflammatory cell infiltration was scored as grade 0, 0%; grade 1, 1%-20%; grade 2, 21%-40%; grade 3, 41%-60%; grade 4, 61%-80%; and grade 5, 81%-100%. To assay neutrophil infiltration, the tissue sections were stained with anti-Ly-6G antibody (ab25377; Abcam).

Apoptosis in human and mouse colon tissues was detected using the terminal deoxynucleotidyl transferase dUTP nick end labeling (TUNEL) assay kit (Beyotime, Shanghai, China) as described previously 31.

### Annexin V-FITC/PI apoptosis assay

Cell apoptosis was detected using the Annexin V-FITC/PI Apoptosis Detection Kit (Beyotime) according to manufacturer's instructions.

### Immunofluorescence

Immunofluorescence was performed as described previously 16. Primary antibodies used in this study included anti-γ-H2AX antibody (2577; Cell Signaling Technology) and anti-double stranded DNA monoclonal antibody (MAB1293; Millipore).

### Real-time RT-PCR

Real-time RT-PCR was performed as described previously 16. The primer sequences were as follows, Dicer (human), 5'-TCCACGAGTCACAATCAACACGG-3' and 5'-GGGTTCTGCATTTAGGAGCTAGATGAG-3'; IL-6 (human), 5'-CAATCTGGATTCAATGAGGAGAC-3' and 5'-CTCTGGCTTGTTCCTCACTACTC-3'; *GAPDH* (human), 5'-ATGACATCAAGAAGGTGGTG-3' and 5'-CATACCAGGAAATGAGCTTG-3'; Dicer (mouse), 5'-GCCAAGAAAATACCAGGTTGAGC-3' and 5'-GCGATGAACGTCTTCCCTGAG-3';* GAPDH* (mouse), 5'-ACGGCCGCATCTTCTTGTGCA-3' and 5'-ACGGCCAAATCCGTTCACACC-3'. To evaluate miR-215 expression, real-time RT-PCR was performed using the bulge-loop miRNA qPCR primer set (RiboBio, Guangzhou, China) according to manufacturer's instructions.

### Dual-luciferase assays

Dual-luciferase assays were performed using the Dual-Luciferase Reporter Assay System (Promega) as described previously 31.

### Comet assay

Comet assay was performed as described previously 16, 31.

### Detection of 8-hydroxydesoxyguanosine

The colon tissues were homogenized with PBS and centrifuged (12000 × g for 15 min at 4 °C). The supernatants were collected to determine the total protein concentration using a BCA protein assay kit (Beyotime). The levels of 8-Hydroxydesoxyguanosine (8-OHdG) in the supernatants were measured using the Enzyme-Linked Immunosorbent Assay Kit For 8-OHdG (Cloud-Clone Corp., Houston, TX) according to the manufacturer's instructions. The results are expressed as μg of 8-OHdG per mg of total protein (μg/mg protein).

### Statistical analysis

All experimental data are presented as means ± SEM of at least three independent experiments. The number of mice per group is indicated in the figures, and significant differences between groups were determined using Student's *t*-test when variances were equal. When variances were unequal, Welch's *t*-test was used. The correlation between two variables was assessed by Spearman correlation analysis. T-tests were performed using GraphPad Prism 5.0 software (GraphPad Software Inc., La Jolla, CA) and Spearman correlation analysis was performed using SPSS 22.0 software (IBM, Armonk, NY). P-values < 0.05 were considered statistically significant.

## Results

### Dicer is downregulated in inflamed colon tissues before malignancy occurs

**Figure 1:**
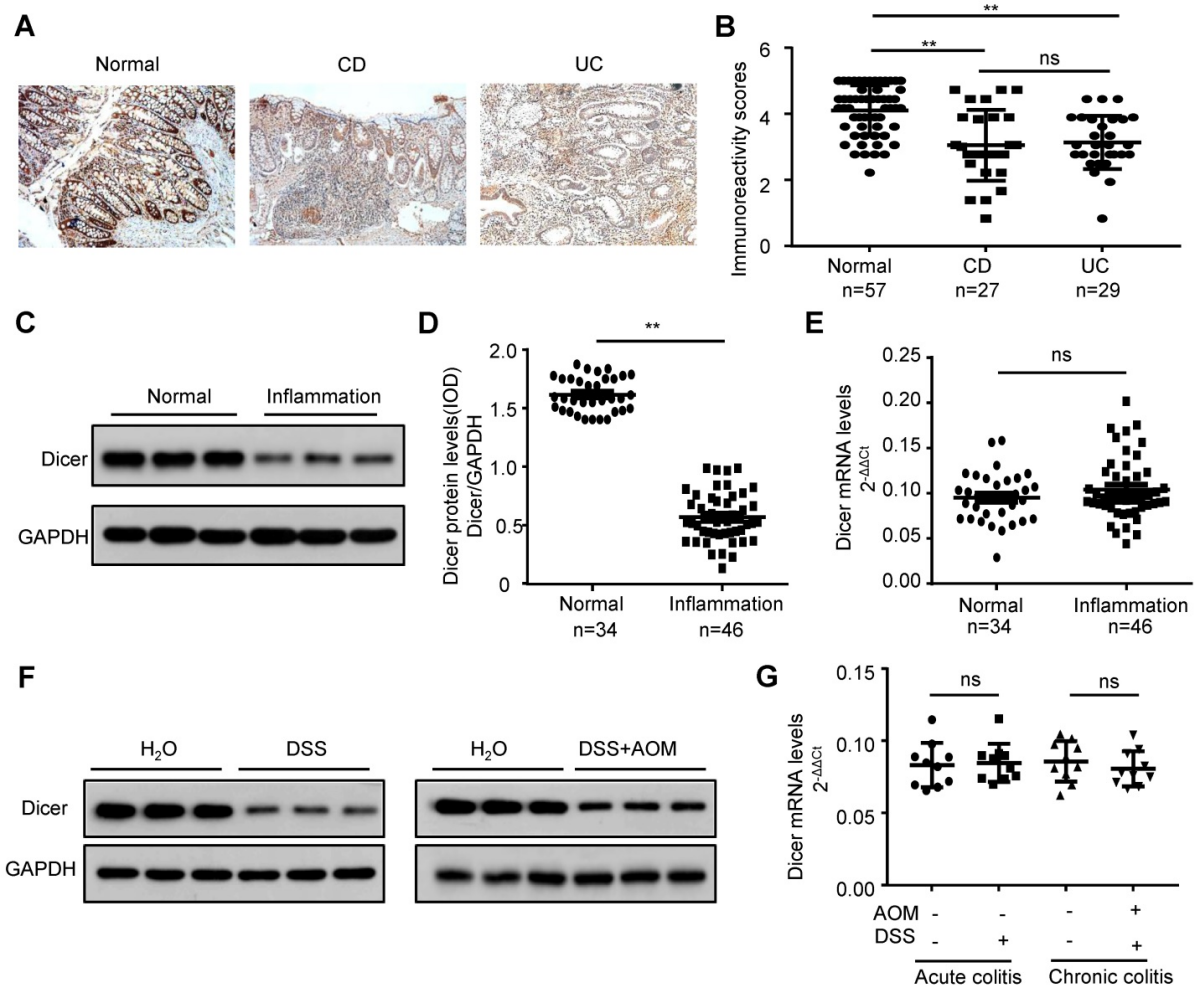
** Decreased Dicer expression in inflamed colon tissues.** (A, B) Immunohistochemistry of Dicer expression in 56 inflamed colon tissues and 57 normal colon tissues. Representative immunohistochemistry images (A) and semi-quantitative evaluation (B) of Dicer protein expression. (C-E) Analysis of Dicer expression in 46 inflamed colon tissues and 34 normal colon tissues. Representative western blotting images of Dicer protein levels in three normal colon tissues and three inflamed colon tissues (C). Dicer and GAPDH protein levels were determined via densitometry using ImageJ and are represented as IOD (D). Dicer mRNA levels were determined by real-time RT-PCR (E). (F, G) Dicer expression in colon tissues derived from control mice, DSS-induced acute, or AOM/DSS-induced chronic colitis mice was determined by western blotting (F) and real-time RT-PCR (n = 10 mice per group) (G). Data represent the means ± SEM. **P < 0.01. ns, not significant. AOM: azoxymethane; CD: Crohn's disease; DSS: dextran sulfate sodium; IOD: integrated optical density; UC: ulcerative colitis.

To investigate whether Dicer is downregulated in inflamed colon tissues before a malignant change occurs, we first examined Dicer expression in paraffin-embedded colon tissues from 56 patients with IBD (27 Crohn's disease and 29 ulcerative colitis) and 57 controls. Immunochemistry revealed that Dicer was downregulated in inflamed colon tissues compared with control colon tissues (Figure 1A-B). Using frozen inflamed colon tissues from another 46 patients with IBD and 34 controls, we found that Dicer was downregulated at the protein level, but not at the mRNA level (Figure 1C-E). Moreover, we found that Dicer was also downregulated at the protein level but not at the mRNA level in inflamed colon tissues derived from DSS-induced acute or AOM plus DSS-induced chronic colitis mouse models (Figure 1F-G). Collectively, these findings suggest that Dicer expression is downregulated in inflamed colon tissues before malignancy occurs.

### Oxidative stress represses Dicer expression in inflamed colon tissues

**Figure 2:**
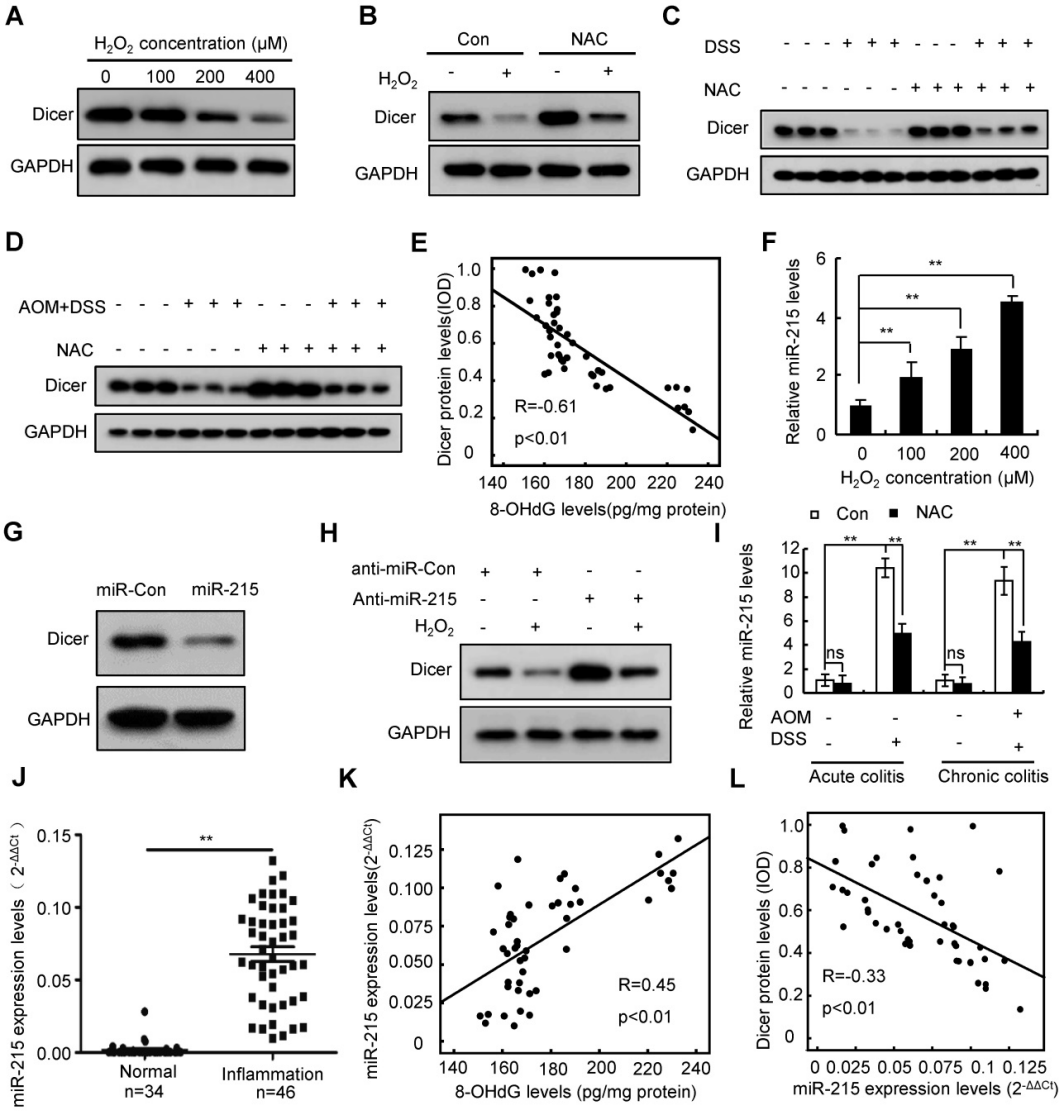
** Oxidative stress represses Dicer expression in inflamed colon tissues by inducing miR-215 expression.** (A) FHC cells were treated with different doses of H_2_O_2_, Dicer protein level was determined 24 h after treatment. (B) FHC cells were treated with 400 µM H_2_O_2_ and 2 mM NAC; Dicer protein levels were determined 24 h after treatment. (C, D) Dicer expression in colon tissues derived from DSS-induced acute (C) or AOM/DSS-induced chronic (D) colitis mouse models with or without NAC treatment was determined by western blotting. (E) Correlation between 8-OHdG levels and Dicer levels in 46 inflamed colon tissues. (F) FHC cells were treated with different doses of H_2_O_2_ for 24 h, and the level of miR-215 was quantified. (G) FHC cells were transfected with miR-215 mimics, and Dicer protein levels were determined 48 h post-transfection. (H) FHC cells were transfected with miR-215 inhibitors, and 400 µM H_2_O_2_ was added to the culture medium 24 h after transfection. Dicer protein levels were determined 24 h after H_2_O_2_ treatment. (I) miR-215 levels were quantified in colon tissues derived from acute or chronic colitis mouse models treated with or without NAC; n = 8 mice per group. (J) miR-215 levels were quantified in 34 normal control colon tissues and 46 inflamed colon tissues. (K) Correlation between 8-OHdG levels and miR-215 levels in 46 inflamed colon tissues. (L) Correlation between miR-215 levels and Dicer protein levels in 46 inflamed colon tissues. Data represent the means ± SEM. **P < 0.01. ns, not significant. AOM: azoxymethane; DSS: dextran sulfate sodium; NAC: N-acetyl-L-cysteine

Inflammatory conditions inevitably lead to oxidative stress 32. To investigate whether inflammation represses Dicer expression via oxidative stress, we treated the human colon epithelial cell line FHC, the human colon myofibroblast cell line CCD-18Co, and the human macrophage cell line THP-1 with hydrogen peroxide (H_2_O_2_). Our results revealed that H_2_O_2_ inhibited Dicer protein, but not mRNA, expression in a dose- and time-dependent manner (Figure 2A and Figure S1A-D). Treatment with N-acetyl-L-cysteine (NAC), an effective antioxidant, partially rescued the H_2_O_2_-induced Dicer downregulation (Figure 2B and Figure S1E). Moreover, administration of NAC to colitis mouse models partially rescued Dicer expression in inflamed colon tissues (Figure 2C-D). As expected, 8-hydroxy-2'-deoxyguanosine (8-OHdG), a biomarker of oxidative stress and oxidative DNA damage 33, was increased in human and mouse inflamed colon tissues compared with control colon tissues (Figure S2). Moreover, we detected an inverse correlation between Dicer protein levels and 8-OHdG levels in human inflamed colon tissues (Figure 2E). Therefore, these results indicate that oxidative stress represses Dicer expression in inflamed colon tissues.

### Oxidative stress represses Dicer expression in inflamed colon tissues by inducing miR-215 expression

Analysis of previously published microarray data 34 revealed that H_2_O_2_ induced miR-215 expression in a time-dependent manner in mouse fibroblasts (Figure S3A). Quantitative RT-PCR confirmed that H_2_O_2_ treatment dose-dependently induced miR-215 expression in FHC, CCD-18Co, and THP-1 cells (Figure 2F and Figure S3B). Targetscan prediction revealed that there are three potential miR-215 binding sites on the 3′-untranslated region (UTR) of Dicer (Figure S3C; top panel). To examine whether miR-215 regulates Dicer expression, we performed a dual luciferase assay using three reporter vectors consisting of the luciferase coding sequence followed by different fragments of the 3′-UTR of Dicer (Figure S3C; bottom panel). As shown in Figure S3D, miR-215 repressed luciferase activity of all three reporters. Transient transfection of miR-215 decreased Dicer protein expression but did not affect Dicer mRNA expression (Figure 2G and Figure S3E-F). miR-215 inhibitors partially rescued Dicer protein but not mRNA expression upon H_2_O_2_ treatment (Figure 2H and Figure S3G-H). Collectively, these findings indicate that H_2_O_2_ treatment represses Dicer expression by inducing miR-215 expression.

We then investigated whether oxidative stress in inflamed colon tissues represses Dicer expression by inducing miR-215 expression. As shown in Figure 2I, miR-215 was upregulated in inflamed colon tissues of colitis mouse models; conversely, administration of NAC partially repressed this miR-215 upregulation. Moreover, miR-215 expression was significantly upregulated and positively correlated with 8-OHdG levels in human inflamed colon tissues (Figure 2J-K). Expression levels of Dicer protein were inversely correlated with miR-215 levels in human inflamed colon tissues (Figure 2L). Together, these results indicate that oxidative stress represses Dicer expression by inducing miR-215 expression.

### Decreased Dicer expression sensitizes cells to oxidative stress-induced DNA damage and apoptosis

As oxidative stress induces DNA damage 32, and Dicer plays important roles in DNA repair 16-21, we examined whether decreased Dicer expression sensitizes cells to oxidative stress-induced DNA damage. Comet assays and immunostaining with γ-H2AX antibody indicated that H2O2 induced more DNA damage in Dicer knockdown cells than in control cells (Figure S4). Cell proliferation and apoptosis assays also revealed that Dicer knockdown sensitized cells to H2O2 treatment (Figure S5A-B). TUNEL staining revealed that apoptosis was increased in inflamed colon tissues (Figure S5C) and that Dicer knockdown further increased apoptosis in DSS-induced inflamed colon tissues (Figure S5D). Interestingly, we found an inverse correlation between expression levels of Dicer protein and apoptosis levels in human inflamed colon tissues (Figure S5E).

### Decreased Dicer expression increases cytosolic DNA and promotes IL-6 expression upon H_2_O_2_ treatment

**Figure 3:**
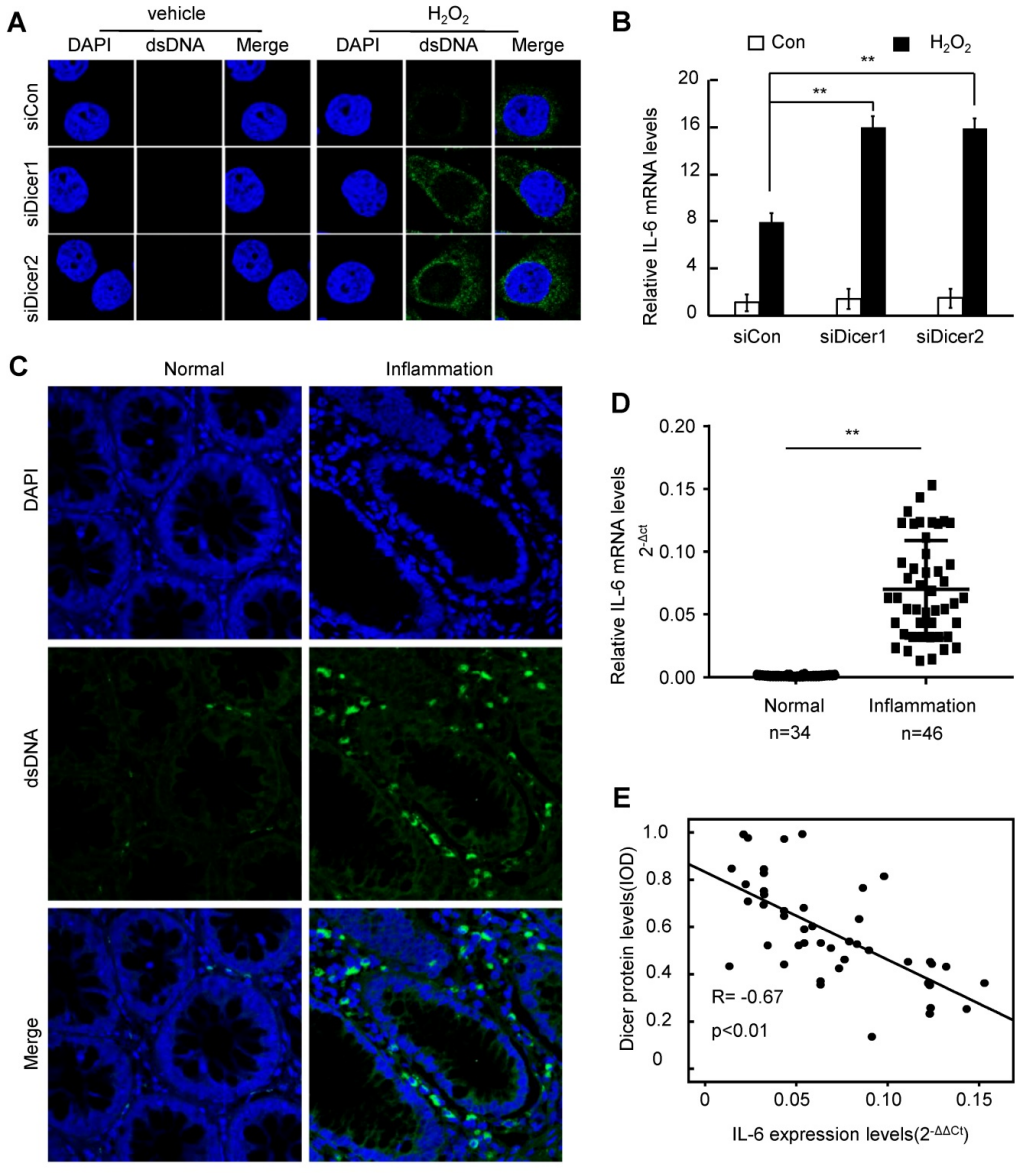
** Decreased Dicer expression leads to increased cytosolic DNA and IL-6 expression after H_2_O_2_ treatment.** (A, B) FHC cells were transfected with control or Dicer siRNAs, and 400 µM H_2_O_2_ was added to the culture medium 24 h post-transfection; cytosolic dsDNA levels (A) as well as IL-6 mRNA levels (B) were determined 24 h after H_2_O_2_ treatment. (C) Representative confocal microscopy image of immunocytochemistry for cytosolic DNA in inflamed and control colon tissues. (D) IL-6 mRNA levels were quantified in 34 normal colon tissues and 46 inflamed colon tissues. (E) Correlation between Dicer protein levels and IL-6 mRNA levels in 46 inflamed colon tissues. Data represent the means ± SEM. **P < 0.01. ns, not significant.

DNA damage leads to accumulation of cytosolic DNA, triggering the production of IL-6, which promotes inflammatory disease and cancer 24, 25, 35. Treatment with H_2_O_2_ increased cytosolic DNA in different cells, and Dicer knockdown further increased the H_2_O_2_-induced cytosolic DNA accumulation (Figure 3A and Figure S6A). Consistently, we found that cytosolic DNA was accumulated in inflamed human colon tissues (Figure 3C). Compared with control cells, Dicer-knockdown cells expressed higher levels of IL-6 upon H_2_O_2_ treatment (Figure 3B and Figure S6B). Furthermore, we found that IL-6 expression was increased in human inflamed colon tissues compared with normal healthy colon tissues (Figure 3D), and an inverse correlation between the expression levels of Dicer protein and the mRNA levels of IL-6 was observed in human inflamed colon tissues (Figure 3E). Thus, these results indicate that decreased Dicer expression promotes the accumulation of cytosolic DNA upon H_2_O_2_ treatment, thereby enhancing H_2_O_2_-induced expression of IL-6.

### Decreased Dicer expression potentiates DSS-induced inflammation in colon tissues and promotes colitis-associated carcinogenesis

Consistent with the finding that heterozygous knockout of Dicer in intestinal epithelial cells increases inflammatory cell infiltration in colon tissues 27, we found that Dicer knockdown in the colon tissues of DSS-induced acute colitis mouse model promoted inflammation in terms of body weight loss, final colon length, inflammatory cell infiltration and cell apoptosis in colon tissues, and serum IL-6 levels (Figure S7, and Figure S5D). Moreover, Dicer knockdown in the colon tissues of AOM/DSS-induced colitis-associated colon cancer mouse model not only increased the severity of inflammation, but also promoted carcinogenesis (Figure S8).

### Anastrozole, berberine, and pranoprofen enhance Dicer expression and decrease H_2_O_2_-induced IL-6 expression

**Figure 4:**
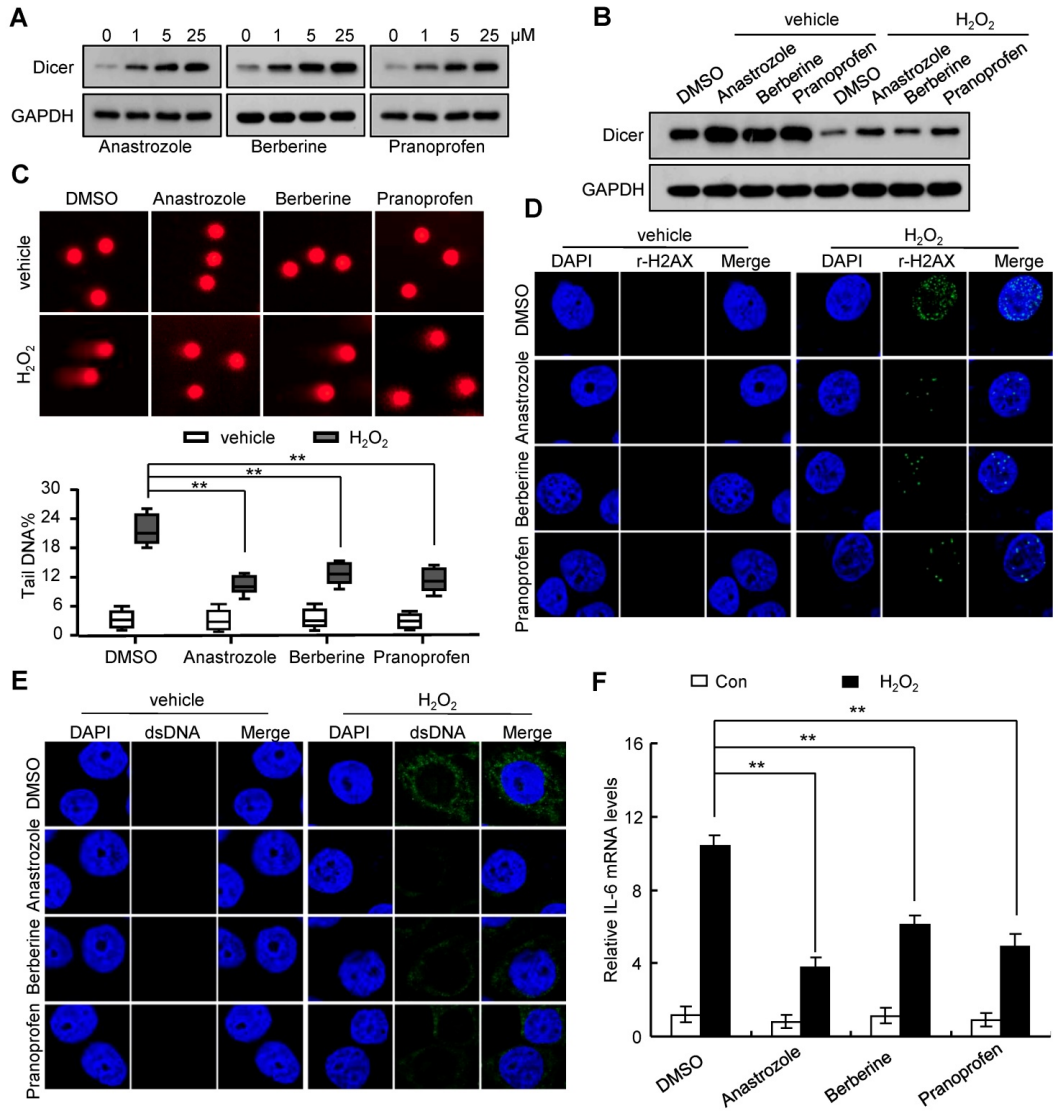
** Anastrozole, berberine, or pranoprofen enhance Dicer expression and decrease H_2_O_2_-induced IL-6 expression.** (A) FHC cells were treated with different doses of anastrozole, berberine, or pranoprofen; Dicer protein levels were determined 24 h after treatment. (B-F) FHC cells were treated with 800 µM H_2_O_2_, 800 µM H_2_O_2_ + 5 µM anastrozole, 800 µM H_2_O_2_ + 5 µM berberine, or 800 µM H_2_O_2_ + 5 µM pranoprofen for 24 h. Dicer protein levels were determined by western blotting (B), DNA damage was assayed by comet assays (C) or immunostaining with γ-H2AX (D), cytosolic dsDNA levels were determined by immunostaining with anti-dsDNA antibody (E), and IL-6 mRNA levels were quantified by real-time RT-PCR (F). Data represent the means ± SEM. **P < 0.01. ns, not significant.

Consistent with our previous report that Dicer overexpression promoted DNA repair 18, we found that Dicer overexpression reduced H_2_O_2_-induced DNA damage, decreased the H_2_O_2_-induced increase in cytosolic DNA, and alleviated the H_2_O_2_-induced IL-6 expression (Figure S9, and Figure S10). By screening a drug library, we found that anastrozole, berberine, and pranoprofen could enhance Dicer expression (Figure 4A and Figure S11A). An increase in Dicer expression induced by anastrozole, berberine, or pranoprofen reduced sensitivity to H_2_O_2_-induced DNA damage (Figure 4B-D and Figure S11B-D). Consequently, treatment with anastrozole, berberine, or pranoprofen decreased the H_2_O_2_-induced increase in cytosolic DNA and IL-6 expression (Figure 4E-F and Figure S12). Silencing Dicer expression partially abrogated these effects of anastrozole, berberine, and pranoprofen (Figure S13, and Figure S14). Collectively, these findings reveal that anastrozole, berberine, or pranoprofen can prevent H_2_O_2_-induced DNA damage by promoting Dicer expression.

### Rescue of Dicer expression in inflamed colon tissues alleviates colitis and represses colitis-associated tumorigenesis

**Figure 5:**
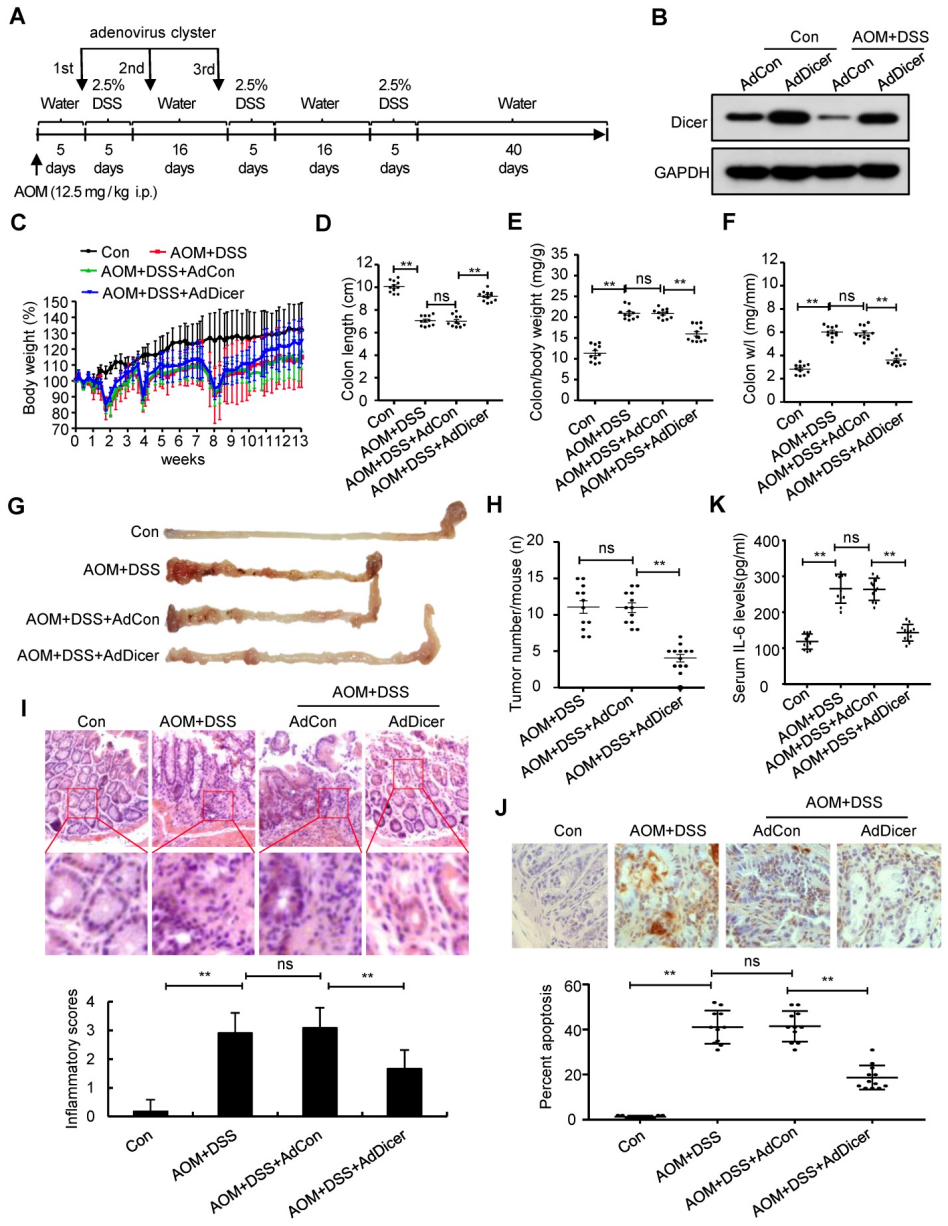
** Dicer overexpression reduces AOM/DSS-induced inflammation in colon tissues and alleviates colitis-associated carcinogenesis.** (A) Schematic of experimental setup: six-week-old male C57BL/6 mice were injected intraperitoneally with 12.5 mg/kg AOM, followed by three cycles of 2.5% DSS treatment. To increase Dicer expression in colon tissues, adenovirus containing the Dicer expression cassette was intrarectally administrated to mice three times. All mice were euthanized 92 days after AOM injection. Mice that received normal drinking water and were not instilled with adenovirus were used as control. (B) Dicer expression in colon tissues was determined by western blotting. (C) Relative body weight curves, (D) colon length, (E) colon/body weight ratio, (F) colon weight/length (w/l) ratio, (G) representative images of mouse gross colon, and (H) tumor numbers in the mouse colorectum. (I) Representative HE-stained colon sections showing inflammatory infiltrate (upper panel) and inflammatory scores (lower panel). (J) Representative images of TUNEL-stained tissue sections (upper panel) and the percentage of apoptotic cells in colon tissues (lower panel). (K) Serum IL-6 levels. Data represent the means ± SEM of at least 11 mice per group. **P < 0.01. ns, not significant. AdCon: control adenovirus; AdDicer: Dicer overexpression adenovirus; AOM: azoxymethane; DSS: dextran sulfate sodium; HE: hematoxylin and eosin; TUNEL: transferase dUTP nick end labeling

We next investigated whether rescue of Dicer expression in inflamed colon tissues alleviates colitis and represses colitis-associated tumorigenesis. Adenovirus-mediated Dicer overexpression in the colon tissues of acute colitis mouse models alleviated inflammation in terms of body weight loss, final colon length, inflammatory cell infiltration, and cell apoptosis, as evidenced by histological analysis and serum IL-6 levels (Figure S15). Dicer overexpression in the colon tissues of chronic colitis mouse models not only reduced the severity of inflammation, but also markedly reduced the number of colon tumors (Figure 5).

**Figure 6:**
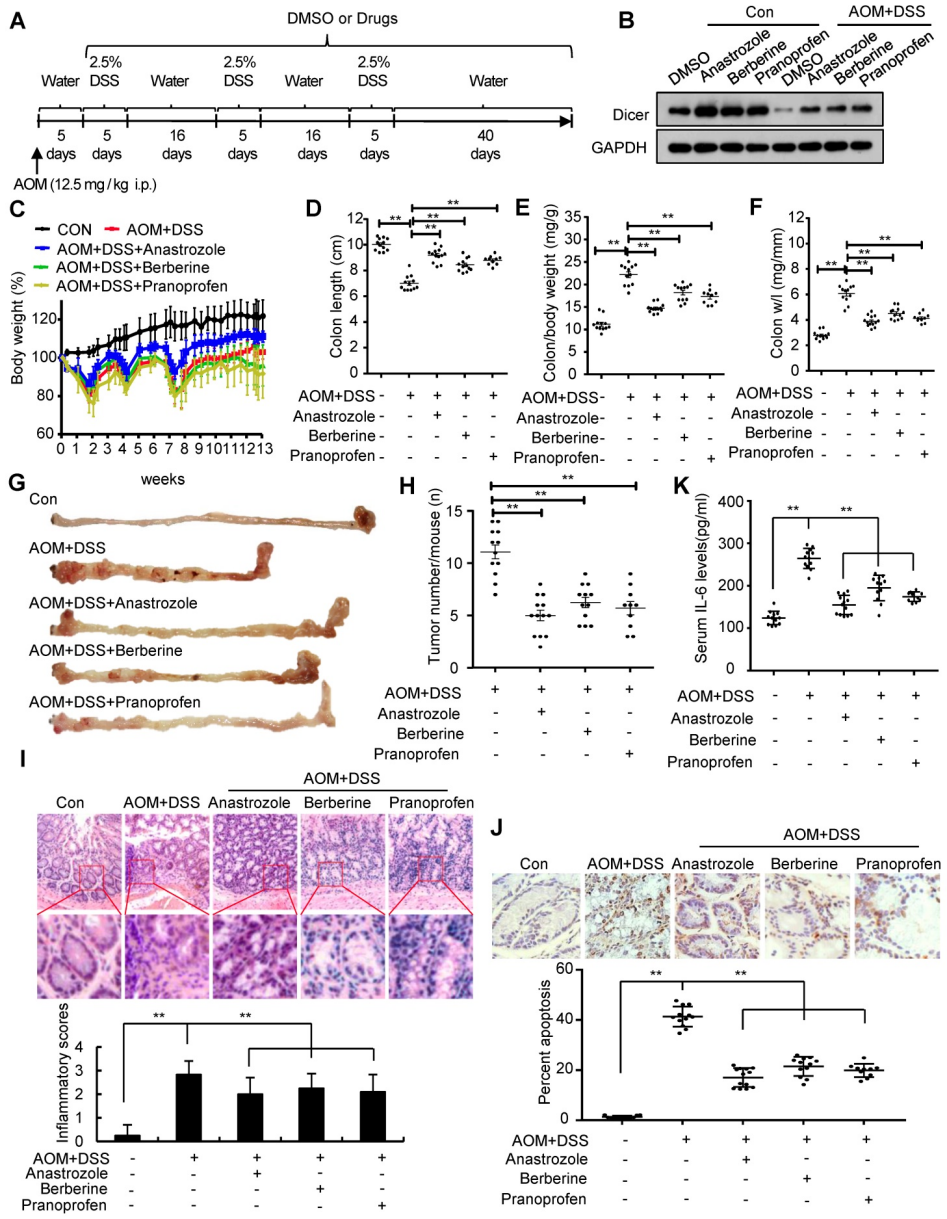
** Upregulation of Dicer expression by anastrozole, berberine, or pranoprofen alleviates inflammation and prevents colitis-associated carcinogenesis.** (A) Schematic of experimental setup: six-week-old male C57BL/6 mice were injected intraperitoneally with 12.5 mg/kg AOM, followed by three cycles of 2.5% DSS treatment. To rescue Dicer expression in inflamed colon tissues, 20 mg/kg anastrozole, 28 mg/kg berberine, or 16 mg/kg pranoprofen was added to the drinking water. (B) Dicer expression in colon tissues was determined by western blotting. (C) Relative body weight curves, (D) colon length, (E) colon/body weight ratio, (F) colon weight/length (w/l) ratio, (G) representative images of mouse gross colon, and (H) tumor numbers in the mouse colorectum. (I) Representative HE-stained colon sections showing inflammatory infiltrate (upper panel) and inflammatory scores (lower panel). (J) Representative images of TUNEL-stained tissue sections (upper panel) and the percentage of apoptotic cells in colon tissues (lower panel). (K) Serum IL-6 levels. Data represent the means ± SEM of at least 8 mice per group. **P < 0.01. HE: hematoxylin and eosin; TUNEL: transferase dUTP nick end labeling

Administration of anastrozole, berberine, or pranoprofen to colitis mouse models rescued Dicer expression in inflamed colon tissues (Figure 6A-B and Figure S16A-B). Pharmacological rescue of Dicer expression in an acute colitis mouse model alleviated inflammation in terms of final colon length, inflammatory cell infiltration, and cell apoptosis in colon tissues, and serum IL-6 levels (Figure S16C-J, and Figure S17). Similar to Dicer overexpression, pharmacological rescue of Dicer expression in a chronic colitis mouse model reduced inflammation severity and colon tumor formation (Figure 6C-J and Figure S18). Silencing Dicer expression in colon tissues partially abrogated the effects of anastrozole, berberine, and pranoprofen on inflammation and inflammation-associated colon cancers (Figure S19, and Figure S20), thereby indicating that these drugs alleviate colitis and prevent colitis-associated colon cancers via upregulating Dicer expression.

## Discussion

In this study, we found that oxidative stress in inflamed colon tissues repressed Dicer expression by inducing miR-215 expression, and that decreased Dicer expression sensitized cells to oxidative stress-induced DNA damage and promoted IL-6 expression in inflamed colon tissues. These findings indicate that inflammation represses Dicer expression, and decreased Dicer expression further exaggerates inflammation. Moreover, we demonstrated that adenovirus-mediated Dicer overexpression in inflamed colon tissues alleviates inflammation and represses colitis-associated tumorigenesis. Collectively, our findings suggest that Dicer downregulation in inflamed tissues drives a local auto-amplification loop that leads to uncontrolled inflammation, which may promote cancer initiation and progression 26.

miR-215 is a p53 target that is upregulated in response to DNA damage 36. It functions as a tumor suppressor through various mechanisms, e.g., inducing cell cycle arrest and promoting cancer stem cell differentiation 36-38. Low miR-215 expression in renal cell carcinoma tissues is associated with a significantly reduced disease-free survival time 38. However, other studies have demonstrated that miR-215 has oncogenic functions as it targets tumor suppressors 39, 40 and that a high expression of miR-215 in colon cancer tissues is associated with poor overall survival 41. Our findings revealed that miR-215 might function as an oncogene in inflammation-driven carcinogenesis by repressing Dicer expression. The effect of oxidative stress on miR-215 expression was multifaceted. As DNA damage induces miR-215 transcription 36, oxidative stress can induce DNA damage and therefore promote miR-215 transcription. Consistent with this, we found that H2O2-treatment not only increased the levels of mature miR-215, but also increased the levels of pri-miR-215 and pre-miR-215 (Figure S21), suggesting that oxidative stress may induce miR-215 transcription. However, given that miR-215 can repress Dicer expression, and Dicer is essential for the procession of pre-miR-215 into mature miR-215, Dicer downregulation may facilitate a feedback to prevent excessive miR-215 upregulation upon oxidative stress. Indeed, we found that the increase of mature miR-215 was less than that of pri-miR-215 and pre-miR-215 upon H_2_O_2_-treatment (Figure S21). Moreover, we found that berberine, anastrozole, and pranoprofen did not affect expression and function of miR-215 (Figure S22), indicating that these drugs promote Dicer expression via a miR-215-independent mechanism.

Anastrozole is a nonsteroidal aromatase inhibitor that can reversibly bind to the aromatase enzyme and block the conversion of androgens to estrogens, and is used to treat or prevent breast cancer 42. In the present study, we found that anastrozole could be used to alleviate inflammation in colon tissues and prevent colitis-associated colon cancer by enhancing Dicer expression. Although the molecular mechanisms underlying how anastrozole induces Dicer expression remains to be elucidated, the mechanisms are not likely to be dependent on the activity of aromatase because letrozole, another nonsteroidal aromatase inhibitor, does not affect Dicer expression (Figure S21).

Berberine, a natural plant product, has been shown to lower blood glucose and lipid levels and increase insulin sensitivity in numerous clinical trials 43, 44. Preclinical data showed that berberine exerts anti-inflammatory and anti-cancer activities via different mechanisms 43, 44. In this study, we uncovered a new molecular mechanism underlying the anti-inflammatory and anti-cancer activities of berberine, which alleviates oxidative stress-induced DNA damage by promoting Dicer expression, thereby relieving inflammation, and repressing inflammation-driven carcinogenesis. Our finding is consistent with that of a previous study wherein berberine repressed single-strand DNA cleavage induced by H_2_O_2_ and cytochrome *c* 45. However, this is in contrast with another report where berberine was found to repress homologous recombination repair and induce DNA damage 46.

Pranoprofen, a nonsteroidal anti-inflammatory drug used in ophthalmology, inhibits cyclooxygenase and reduces the formation of prostaglandins 47. We found that pranoprofen alleviates DNA damage upon oxidative stress by promoting Dicer expression, thereby relieving inflammation, and preventing colitis-associated carcinogenesis. Although we have not yet elucidated the molecular mechanisms underlying pranoprofen induction of Dicer expression, the mechanisms may not be dependent on the activity of cyclooxygenase because other cyclooxygenase inhibitors, including amfenac sodium monohydrate, naproxen, and rofecoxib, do not affect Dicer expression (Figure S23). Therefore, our findings uncovered a cyclooxygenase-independent anti-inflammatory activity of pranoprofen.

Although all the drugs tested, including anastrozole, berberine and pranoprofen, have been shown to alleviate inflammation in terms of final colon length, inflammatory cell infiltration and cell apoptosis in colon tissues, and serum IL-6 levels, their effects on mouse body weight were different, i.e., anastrozole prevented colitis-induced body weight loss, while berberine and pranoprofen had minimal effects on colitis-induced body weight loss (Figure 6C and Figure S16C, S17A, S18A). In a previous study, berberine was also shown to boost metabolism, improve glucose tolerance, and reduce body weight without altering food intake in db/db mice 48. Given that adenovirus-mediated Dicer overexpression in inflamed colon tissues rescued colitis-induced body weight loss (Figure 5C, and Figure S15C), berberine may both positively and negatively regulate body weight of colitis mice as it can rescue colitis-induced body weight loss by increasing Dicer expression and can decrease body weight by promoting metabolism. Pranoprofen can also positively and negatively regulate body weight of colitis mice as it can prevent colitis-induced body weight loss by increasing Dicer expression and can decrease body weight by reducing food and water intake (Figure S24). Pranoprofen frequently causes gastrointestinal side effects 49, however, and may not be a good candidate for treating colitis. Therefore, further research or clinical trials should be conducted to test the efficacy of anastrozole and berberine in colitis treatment and colitis-associated colon cancer prevention.

## Conclusion

In summary, we found that Dicer was downregulated in inflamed colon tissues before malignancy occurred. Decreased Dicer expression increased cytosolic DNA and promoted IL-6 expression upon oxidative stress. These findings suggest that Dicer downregulation in inflamed tissues drives a local auto-amplification loop that leads to uncontrolled inflammation, which may promote cancer initiation and progression. Moreover, we found that rescue of Dicer expression in inflamed colon tissues by berberine, anastrozole, or pranoprofen alleviated colitis and prevented colitis-associated tumorigenesis. Our study may shed light on potential colitis treatment and colitis-associated colon cancer prevention strategies.

## Supplementary Material

Supplementary figures.

## Abbreviations

8-OHdG: 8-hydroxy-2'-deoxyguanosine
AOM: azoxymethane
DAB: 3,3′-diamino-benzidine
DSS: dextran sulfate sodium
HE: hematoxylin and eosin
HRP: horseradish peroxidase
H_2_O_2_: hydrogen peroxide
IBD: inflammatory bowel diseases
IL-6: interleukin-6
IOD: integrated optical density
NAC: N-acetyl-L-cysteine
PBS: phosphate-buffered saline
SRP: signal recognition particle
tsRNA: tRNA-derived small RNA
TUNEL: transferase dUTP nick end labeling
UTR: untranslated region
