# Supplementary material for: Rescuing Dicer expression in inflamed colon tissues alleviates colitis and prevents colitis-associated tumorigenesis
Source: Theranostics. 2020 Apr 27;10(13):5749–62. doi: 10.7150/thno.41894 (PMC7254990; doi:10.7150/thno.41894)
Supplement: Supplementary file 1 — Supplementary figures. [file thnov10p5749s1.pdf]

Rescuing Dicer expression in inflamed colon tissues alleviates colitis and prevents colitis-associated tumorigenesis

Supplementary Figure Legends

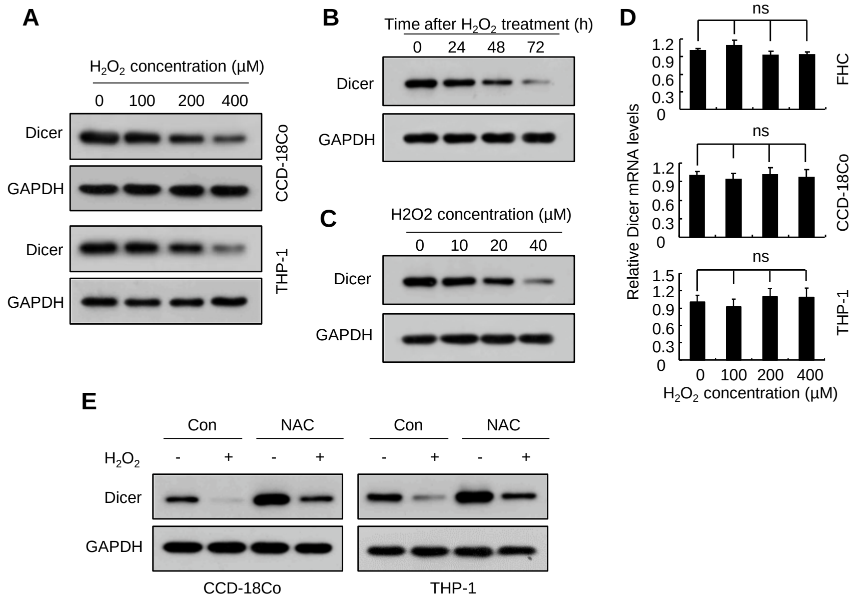

Figure S1. H_2O_2 treatment dose- and time-dependently represses Dicer expression. (A) CCD-18Co and THP-1 cells were treated with different doses (100-400 μM) of H_2O_2; Dicer protein levels were determined 24 h after treatment. (B) FHC cells were treated with 100 μM H_2O_2; Dicer protein levels were determined at different time points. (C) FHC cells were treated with different doses (10-40 μM) of H_2O_2; Dicer protein levels were determined 72 h after treatment. (D) FHC, CCD-18Co, and THP-1 cells were treated with different doses of H_2O_2; Dicer mRNA levels were determined 24 h after treatment. Data represent the means ± SEM. ns, not significant. (E) CCD-18Co and THP-1 cells were treated with 400 μM H_2O_2 and 2 mM NAC; Dicer protein levels were determined 24 h after treatment. NAC: N-acetyl-L-cysteine.

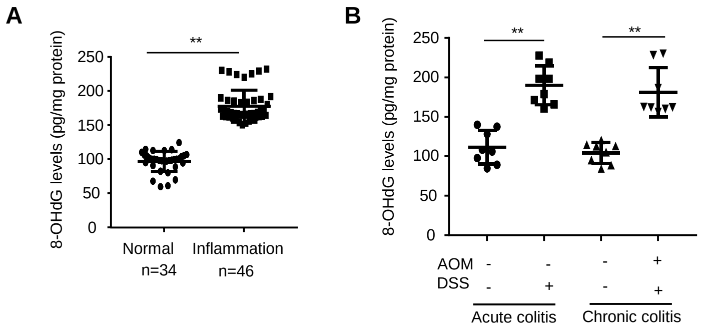

Figure S2. 8-OHdG levels are increased in inflamed colon tissues.

(A) 8-OHdG levels were quantified in 34 normal colon tissues and 46 inflamed colon tissues. (B) 8-OHdG levels were quantified in the colon tissues derived from control mice, DSS-induced acute, or AOM/DSS-induced chronic colitis mice; n = 8 mice per group. Data represent the means ± SEM. **P < 0.01. AOM: azoxymethane; DSS: dextran sulfate sodium.

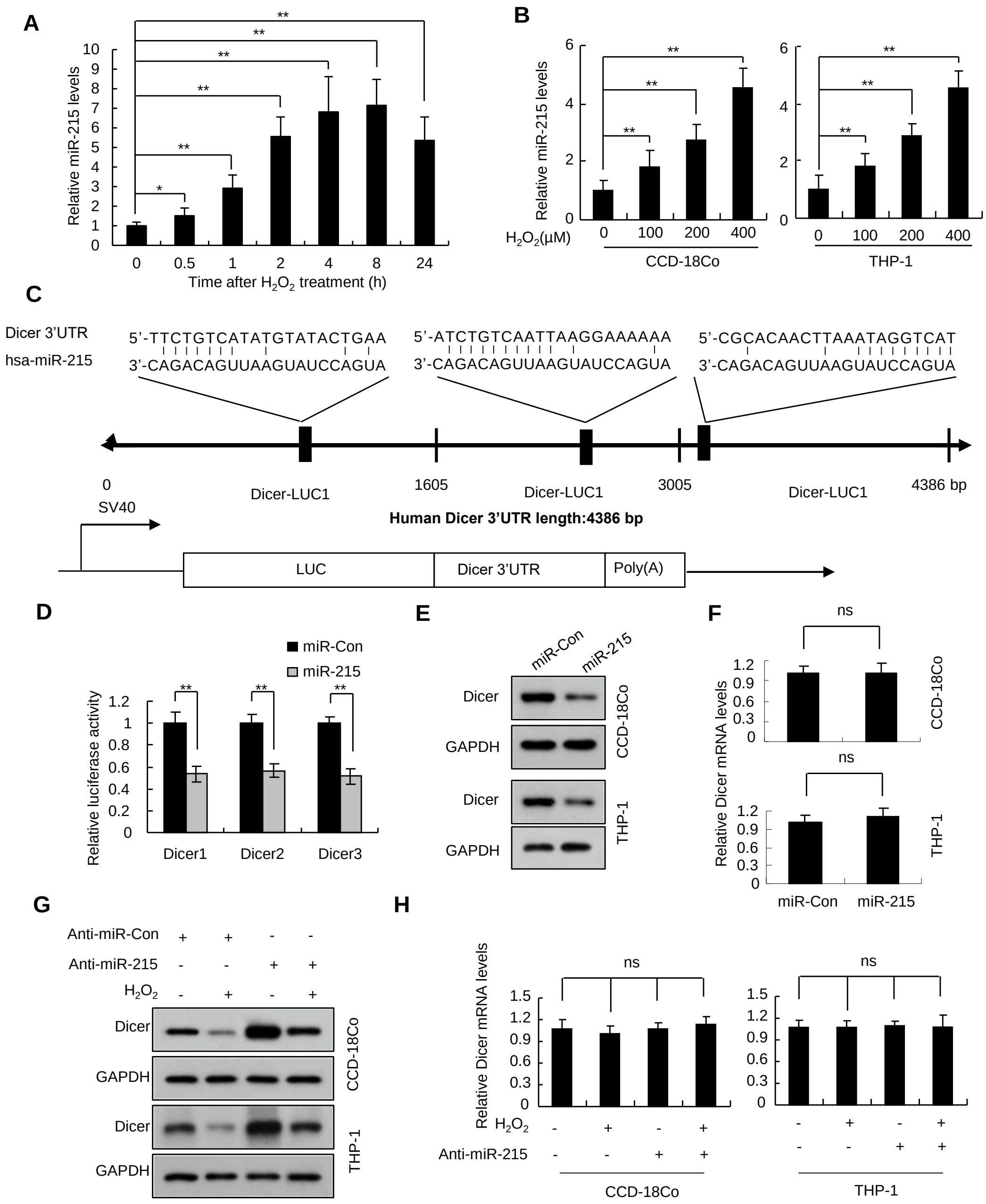

Figure S3. H_2O_2 represses Dicer expression by inducing miR-215 expression.

(A) Mouse fibroblasts were treated with 400 μM H_2O_2; miR-215 expression was determined by microarray at different time points after H_2O_2 treatment. (B) miR-215 levels were quantified in CCD-18Co and THP-1 cells 24h after treatment with different doses of H_2O_2. (C) Predicted miR-215-binding sites in the 3'-UTR of Dicer (top panel) and the pGL3-control luciferase reporter constructs (bottom panel). Dicer UTR, three different fragments of the 3'-UTR of Dicer; poly A, poly(A) tail. (D) FHC cells were cotransfected with miR-

215 mimics and different reporter plasmids along with a pRL-CMV reporter plasmid. Luciferase activity was measured 24 h after transfection. (E, F) CCD-18Co and THP-1 cells were transfected with miR-215 mimics and then Dicer protein (E), and mRNA (F) levels were determined 48 h post-transfection. (G, H) CCD-18Co and THP-1 cells were transfected with miR-215 inhibitors, and 400 μM H_2O_2 was added to the culture medium 24 h after transfection; Dicer protein (G) and mRNA (H) levels were determined 24 h after H_2O_2 treatment. Data represent the means ± SEM. $^{**}P < 0.01$. ns, not significant. Luc: luciferase.

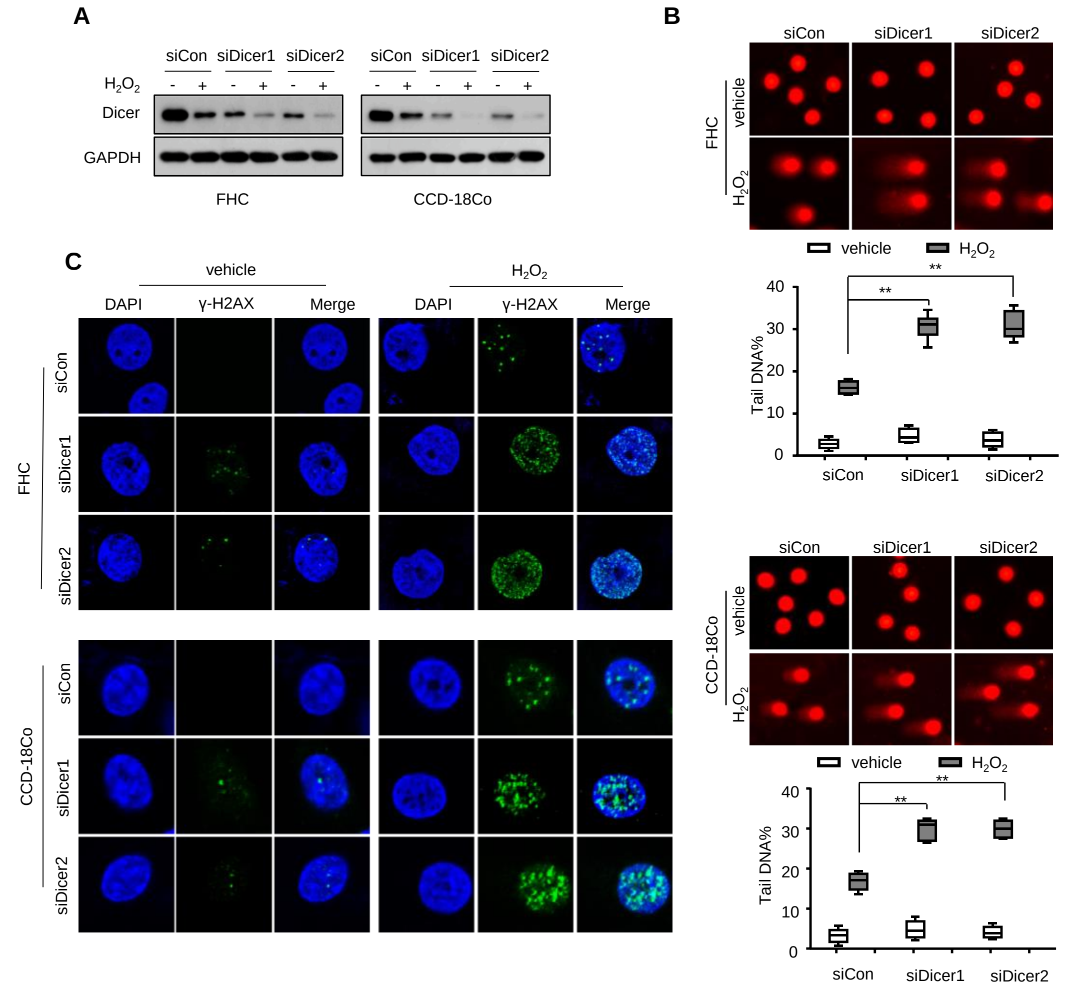

Figure S4. Decreased Dicer expression sensitizes cells to oxidative stress-induced DNA damage.

FHC and CCD-18Co cells were transfected with control or Dicer siRNAs, and 400 μM H_2O_2 was added to the culture medium 24 h post-transfection. (A) Dicer expression was analyzed by western blotting 24 h after H_2O_2 treatment. (B, C) DNA damage was assayed 24 h after H_2O_2 treatment by comet assays (B) or immunostaining with γ-H2AX (C). Data represent the means ± SEM. **$P < 0.01$.

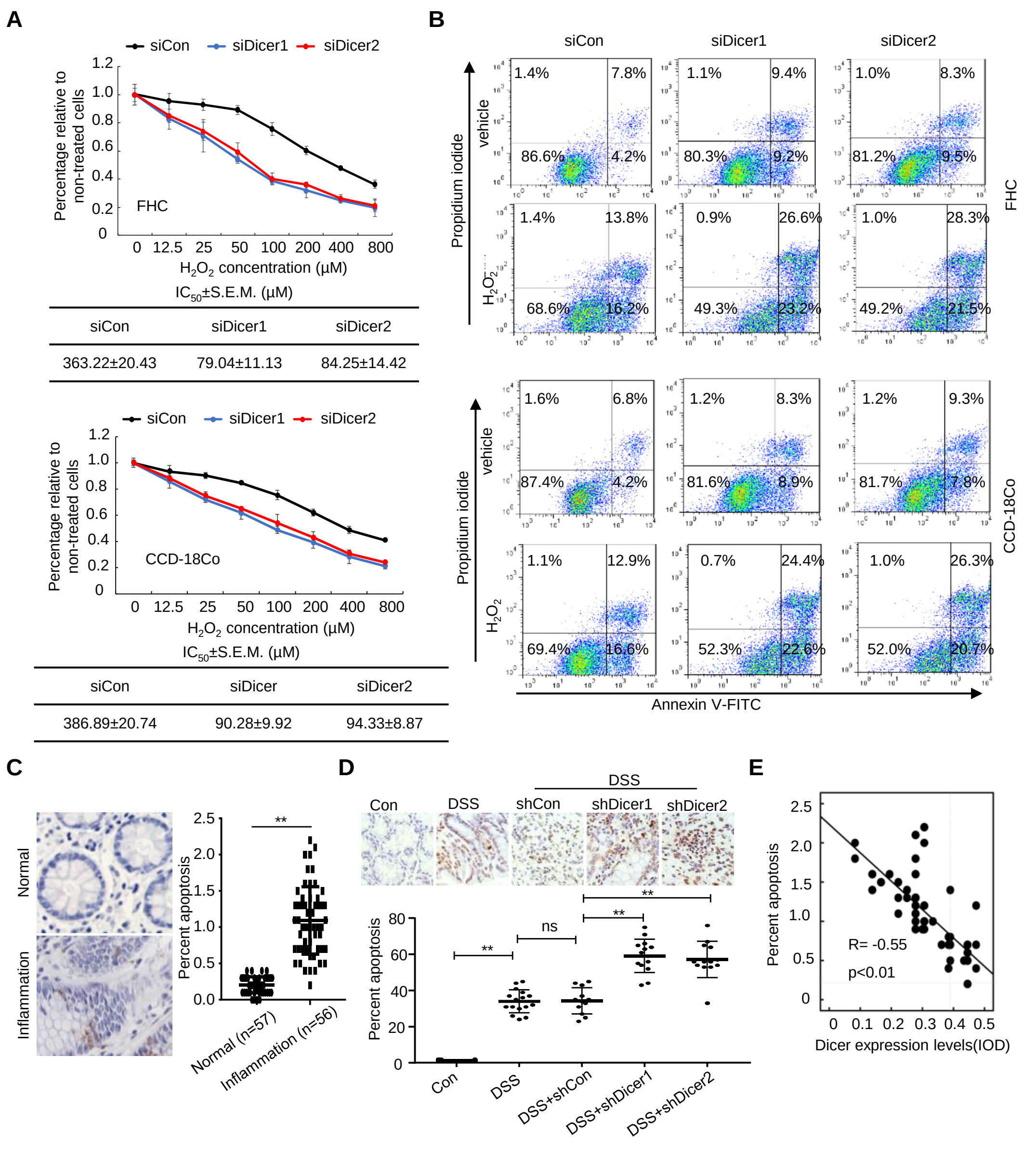

Figure S5. Decreased Dicer expression sensitizes cells to oxidative stress-induced apoptosis.

(A) FHC and CCD-18Co cells were transfected with control or Dicer siRNAs, and different concentrations of H_2O_2 were added to the culture medium 24 h post-transfection. Cell survival was determined using MTT assays 24 h after H_2O_2 treatment. (B) FHC and CCD-18Co cells were transfected with control or Dicer siRNAs, and 400 μM H_2O_2 was added to the culture medium 24 h post-transfection. Apoptosis was assayed using Annexin V-FITC/PI staining 24 h after H_2O_2 treatment. (C) Representative images of TUNEL-stained

human normal colon tissue sections and inflamed colon tissues (left panel) as well as the percentage of apoptotic cells (right panel). (D) Representative images of TUNEL-stained tissue sections (upper panel) and the percentage of apoptotic cells (lower panel) in colon tissues of an acute colitis mouse model; at least 12 mice per group. (E) Correlation between Dicer protein levels and percentage of apoptotic cells in human inflamed colon tissues. Data represent the means ± SEM. **$P < 0.01$. TUNEL: transferase dUTP nick end labeling.

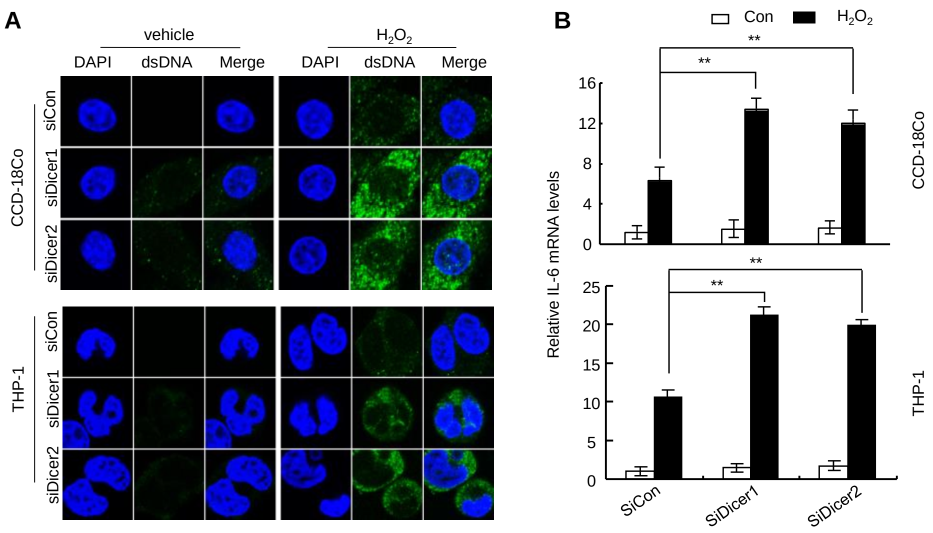

Figure S6. Decreased Dicer expression leads to increased cytosolic DNA and IL-6 expression after H_2O_2 treatment.

(A, B) CCD-18Co and THP-1 cells were transfected with control or Dicer siRNAs, and 400 µM H_2O_2 was added to the culture medium 24 h post-transfection. Cytosolic dsDNA levels (A) as well as IL-6 mRNA levels (B) were determined 24 h after H_2O_2 treatment. Data represent the means ± SEM. $^{**}P < 0.01$.

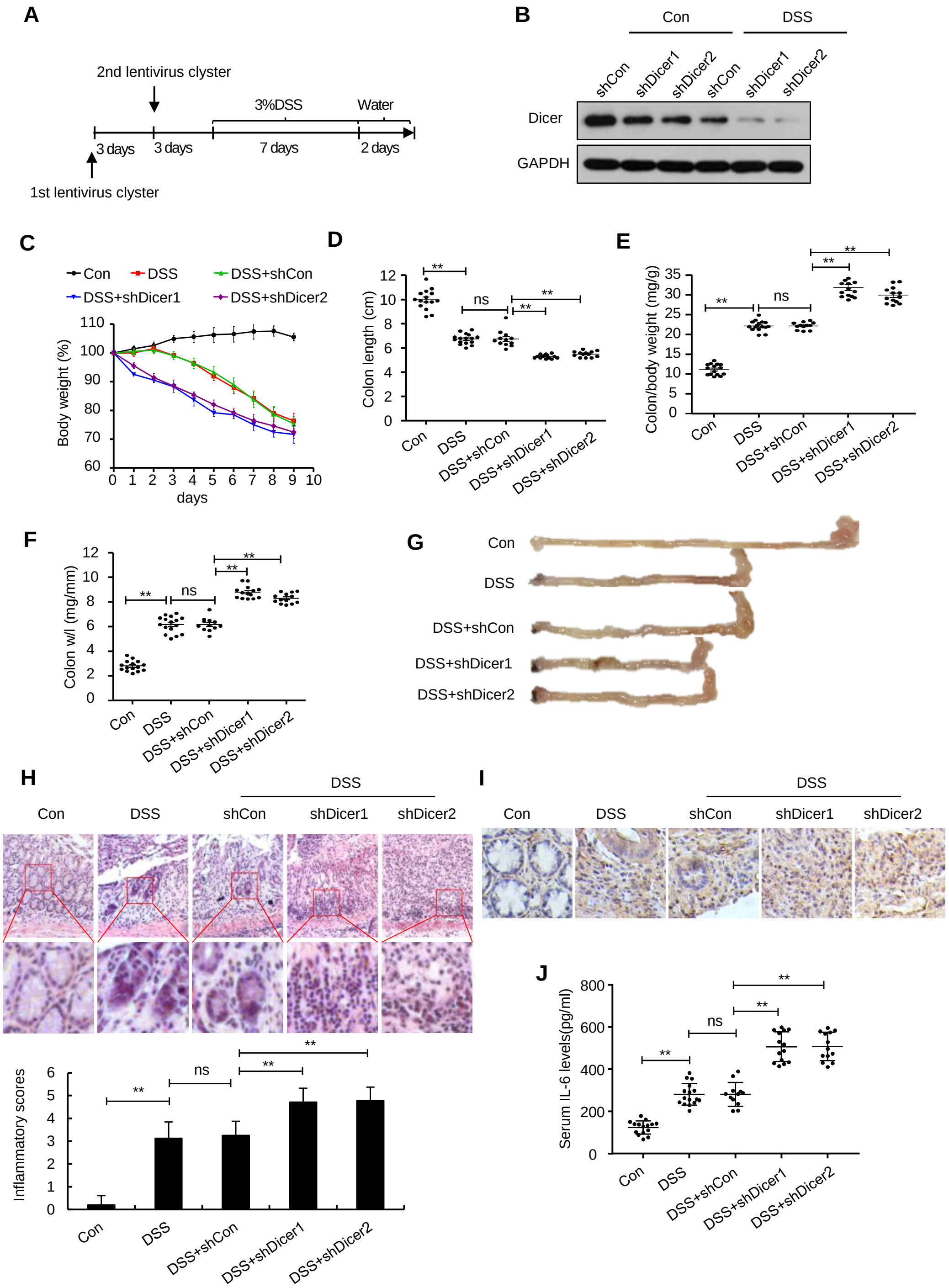

Figure S7. Decreased Dicer expression promotes inflammation in colon tissues of the DSS-induced acute colitis mouse model.

(A) Schematic of experimental setup: five-week-old male C57BL/6 mice were intrarectally instilled with lentivirus expressing Dicer shRNAs or shCon. One week later, acute colitis was induced by oral administration of 3% DSS in drinking water for 7 days followed by 2 days of normal drinking water. All mice were euthanized on day 9. (B) Dicer expression in

colon tissues was determined by western blotting. (C) Relative body weight curves, (D) colon length, (E) colon/body weight ratio, (F) colon weight/length (w/l) ratio, and (G) representative images of mouse gross colon. (H) Representative HE-stained colon sections showing inflammatory infiltrate (upper panel) and inflammatory scores (lower panel). (I) Representative Ly-6G-stained colon sections showing neutrophil infiltrate. (J) Serum IL-6 levels. Data represent the means ± SEM of at least 12 mice per group. **$P < 0.01$. ns, not significant. HE: hematoxylin and eosin.

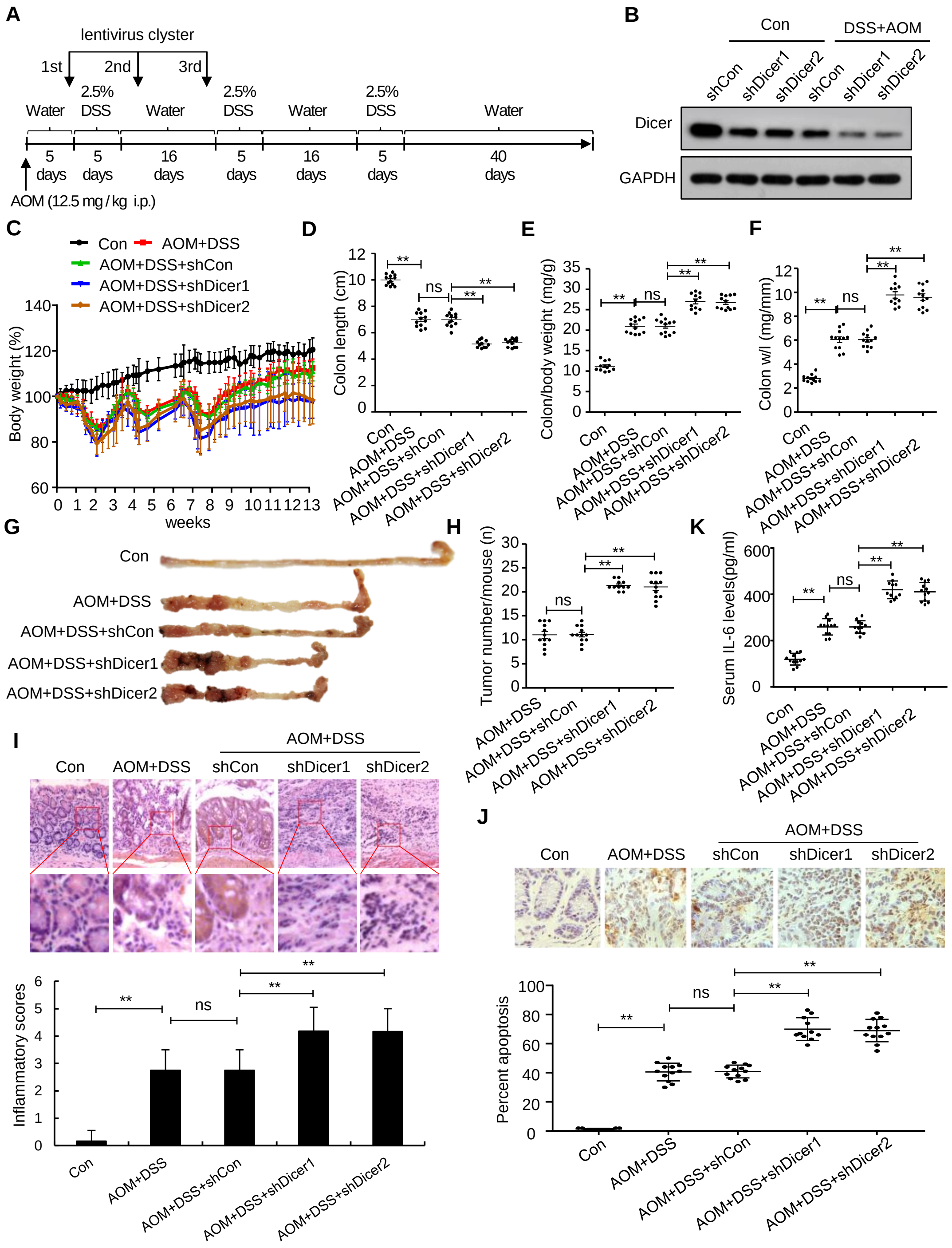

Figure S8. Decreased Dicer expression potentiates AOM/DSS-induced inflammation in colon tissues and promotes colitis-associated carcinogenesis.

(A) Schematic of experimental setup: six-week-old male C57BL/6 mice were injected intraperitoneally with 12.5 mg/kg AOM, followed by three cycles of 2.5% DSS treatment. To knock down Dicer expression in colon tissues, lentivirus containing the Dicer shRNA

expression cassette was intrarectally administrated to mice three times. All mice were euthanized 92 days after AOM injection. Mice that received normal drinking water and were not instilled with lentivirus were used as control. (B) Dicer expression in colon tissues was determined by western blotting. (C) Relative body weight curves, (D) colon length, (E) colon/body weight ratio, (F) colon weight/length (w/l) ratio, (G) representative images of mouse gross colon, and (H) tumor numbers in the mouse colorectum. (I) Representative HE-stained colon sections showing inflammatory infiltrate (upper panel) and inflammatory scores (lower panel). (J) Representative images of TUNEL-stained tissue sections (upper panel) and the percentage of apoptotic cells in colon tissues (lower panel). (K) Serum IL-6 levels. Data represent the means ± SEM of at least 11 mice per group. **$P < 0.01$. ns, not significant. AOM: azoxymethane; DSS: dextran sulfate sodium; TUNEL: transferase dUTP nick end labeling.

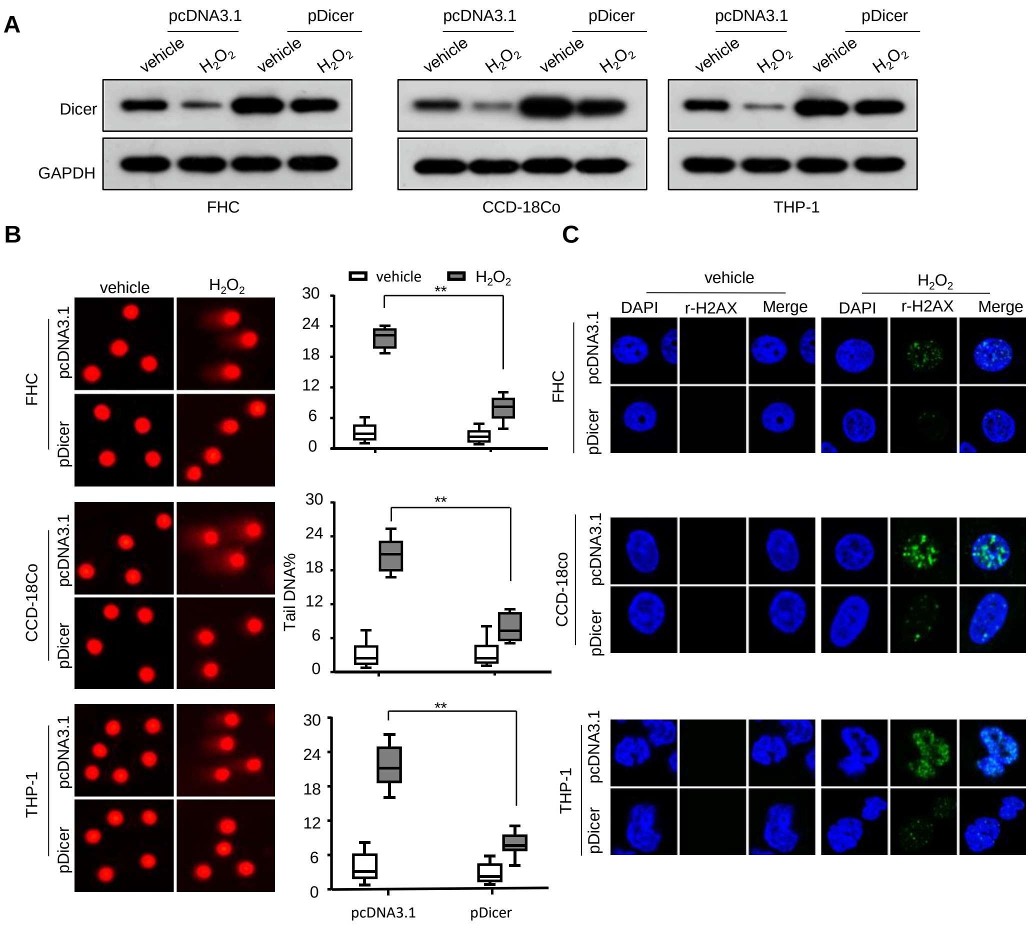

Figure S9. Dicer overexpression reduces H_2O_2-induced DNA damage.

(A–C) FHC, CCD-18Co, and THP-1 cells were transfected with Dicer expression plasmid (pDicer) or pcDNA3.1 as indicated, and 800 μM H_2O_2 was added to the culture medium 24 h post-transfection; 24 h after H_2O_2 treatment, Dicer protein levels were determined by western blotting (A) and DNA damage was assayed by comet assays (B) or immunostaining with γ-H2AX (C). Data represent the means ± SEM. **P < 0.01.

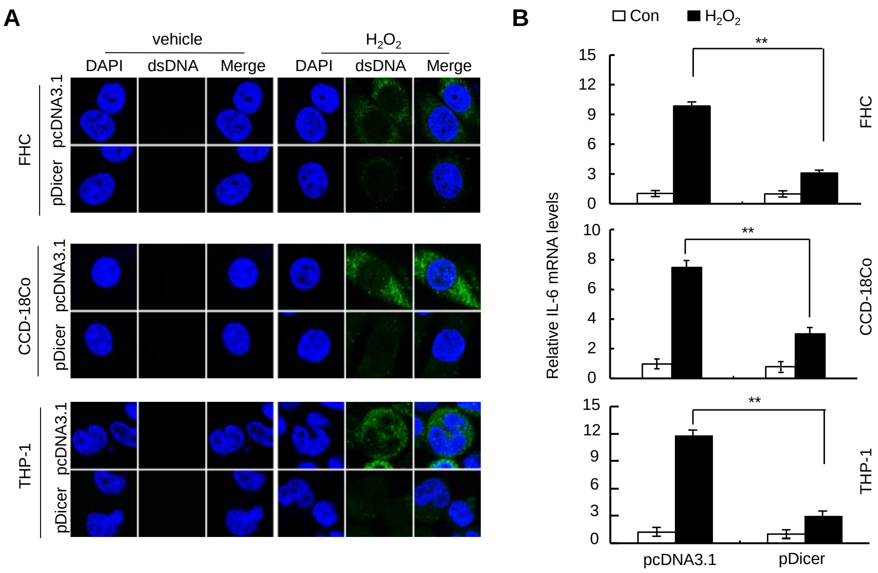

Figure S10. Dicer overexpression attenuates H_2O_2-induced cytosolic DNA accumulation and IL-6 expression.

(A, B) FHC, CCD-18Co and THP-1 cells were transfected with Dicer expression plasmid (pDicer) or pcDNA3.1 as indicated, and 800 µM H_2O_2 was added to the culture medium 24 h post-transfection. Cytosolic dsDNA levels (A) and IL-6 mRNA levels (B) were determined 24 h after H_2O_2 treatment. Data represent the means ± SEM. **P < 0.01.

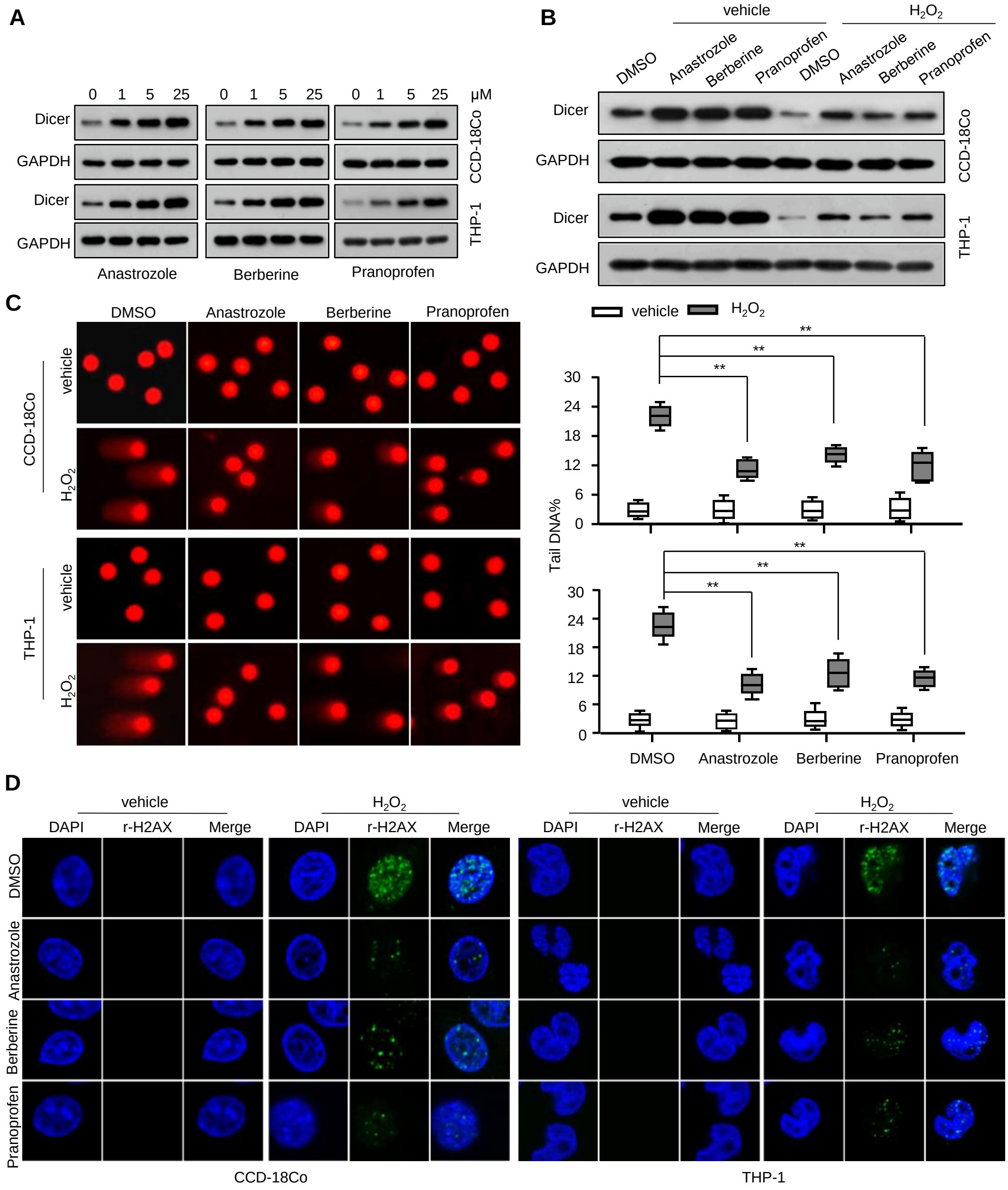

Figure S11. Anastrozole, berberine, or pranoprofen treatment enhances Dicer expression and decreases H_2O_2-induced DNA damage.

(A) CCD-18Co and THP-1 were treated with different doses of anastrozole, berberine, or pranoprofen; Dicer expression levels were determined 24 h after treatment. (B–D) CCD-18Co and THP-1 were treated with 800 μM H_2O_2, 800 μM H_2O_2 + 5 μM anastrozole, 800 μM H_2O_2 + 5μM berberine, or 800 μM H_2O_2 + 5 μM pranoprofen; 24 h after treatment, Dicer expression levels were determined by western botting (B) and DNA damage was

assayed by comet assays (C) or immunostaining with γ-H2AX (D). Data represent the means ± SEM. **P < 0.01.

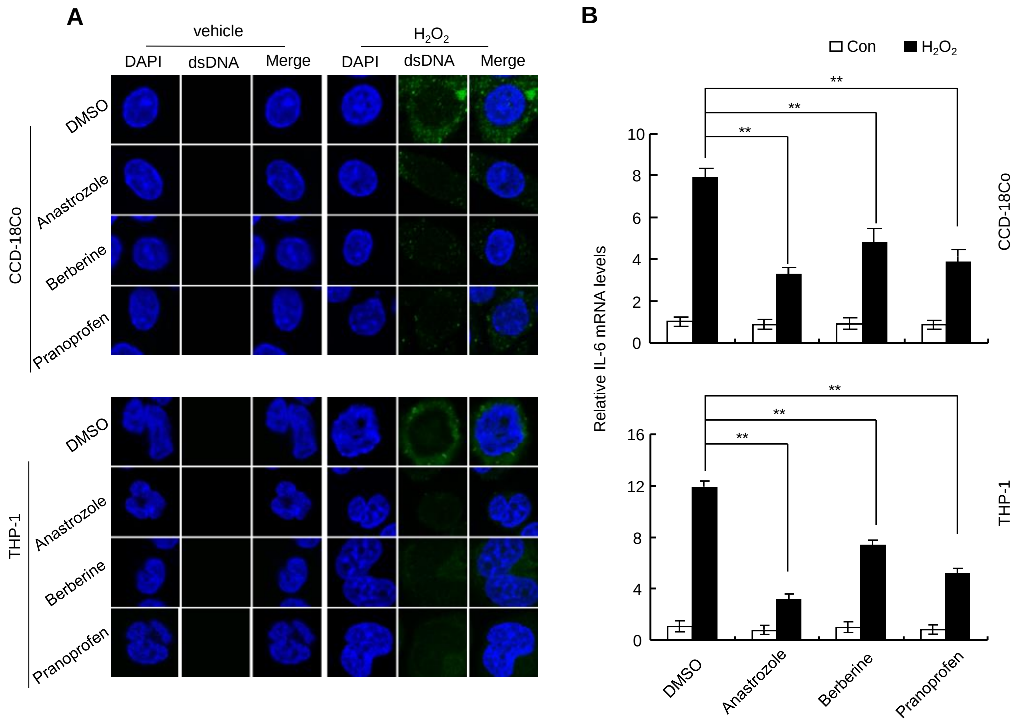

Figure S12. Anastrozole, berberine, or pranoprofen treatment attenuates H_2O_2-induced cytosolic DNA accumulation and IL-6 expression.

(A, B) CCD-18Co and THP-1 were treated with 800 μM H_2O_2, 800 μM H_2O_2 + 5 μM anastrozole, 800 μM H_2O_2 + 5 μM berberine, or 800 μM H_2O_2 + 5 μM pranoprofen. Cytosolic dsDNA levels (A) and IL-6 mRNA levels (B) were determined 24 h after treatment. Data represent the means ± SEM. **$P < 0.01$.

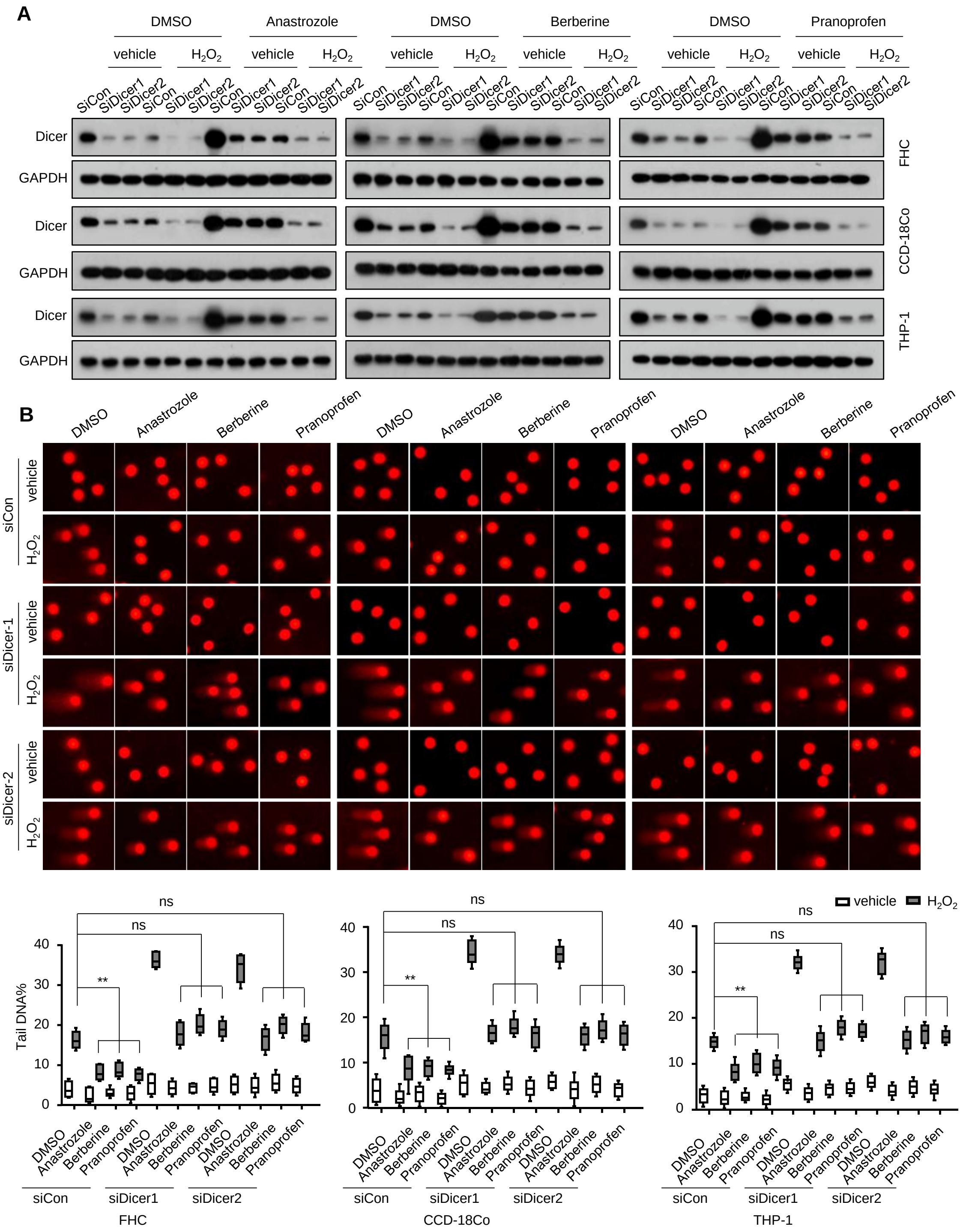
A
DMSO
Anastrozole
DMSO
Berberine
DMSO
Pranoprofen
vehicle
H_2O_2
SiCon
SiDicer1
SiDicer2
Dicer
GAPDH
FHC
CCD-18Co
THP-1
B
Anastrozole
Berberine
Pranoprofen
siCon
siDicer-1
siDicer-2
Tail DNA%
ns
**
siDicer1
siDicer2
vehicle
H_2O_2

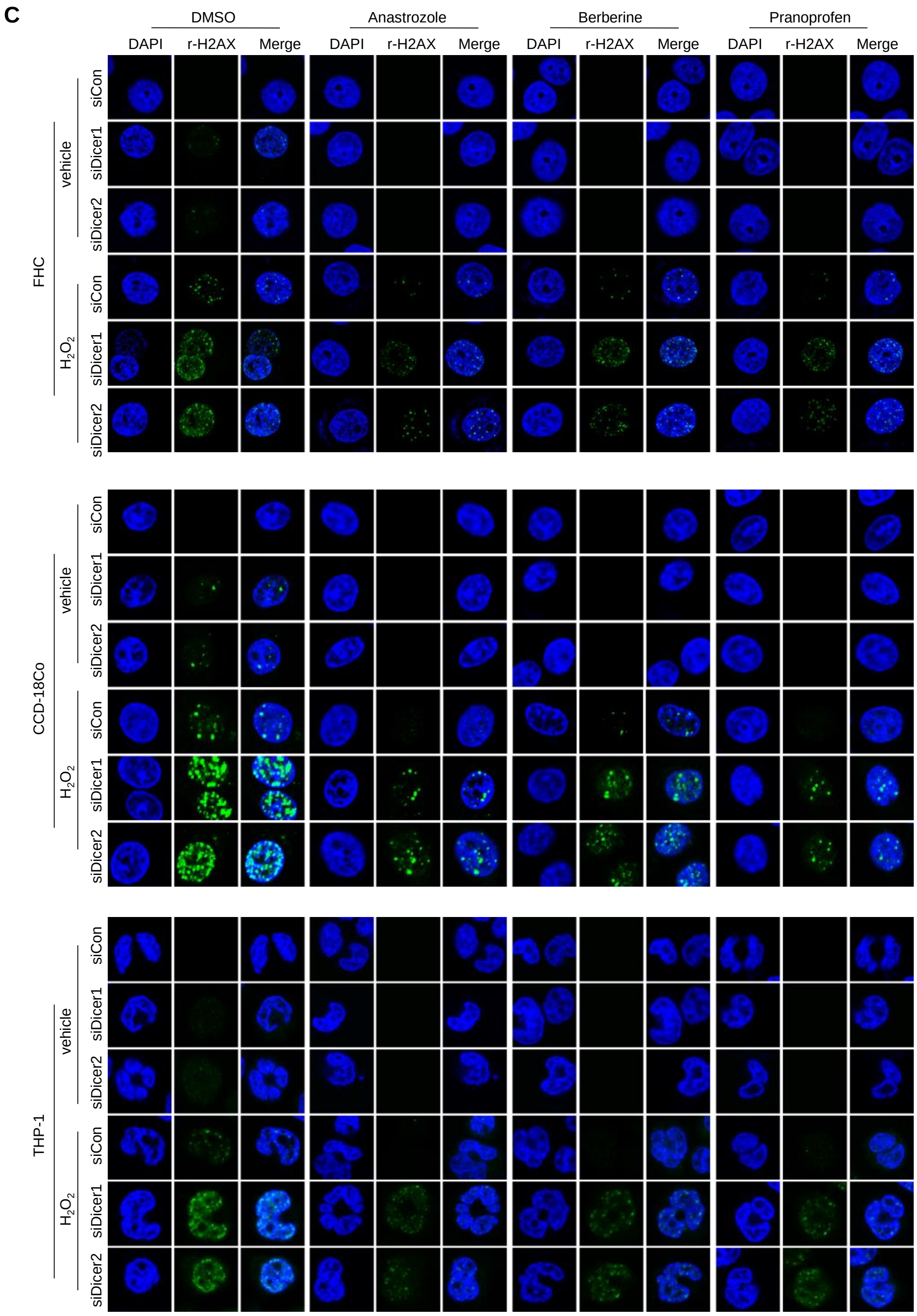

Figure S13. Dicer knockdown partially abrogates the effects of anastrozole, berberine, and pranoprofen on H_2O_2-induced DNA damage.

(A–C) FHC, CCD-18Co, and THP-1 cells were transfected with control or Dicer siRNAs, after which 400 μM H_2O_2, 400 μM H_2O_2 + 5 μM anastrozole, 400 μM H_2O_2 + 5 μM berberine, or 400 μM H_2O_2 + 5 μM pranoprofen were added to the culture medium 24 h

post-transfection; 24 h after treatment, Dicer expression levels were determined by western blotting (A) and DNA damage was assayed by comet assays (B) or immunostaining with γ-H2AX (C). Data represent the means ± SEM. **$P < 0.01$. ns, not significant.

A

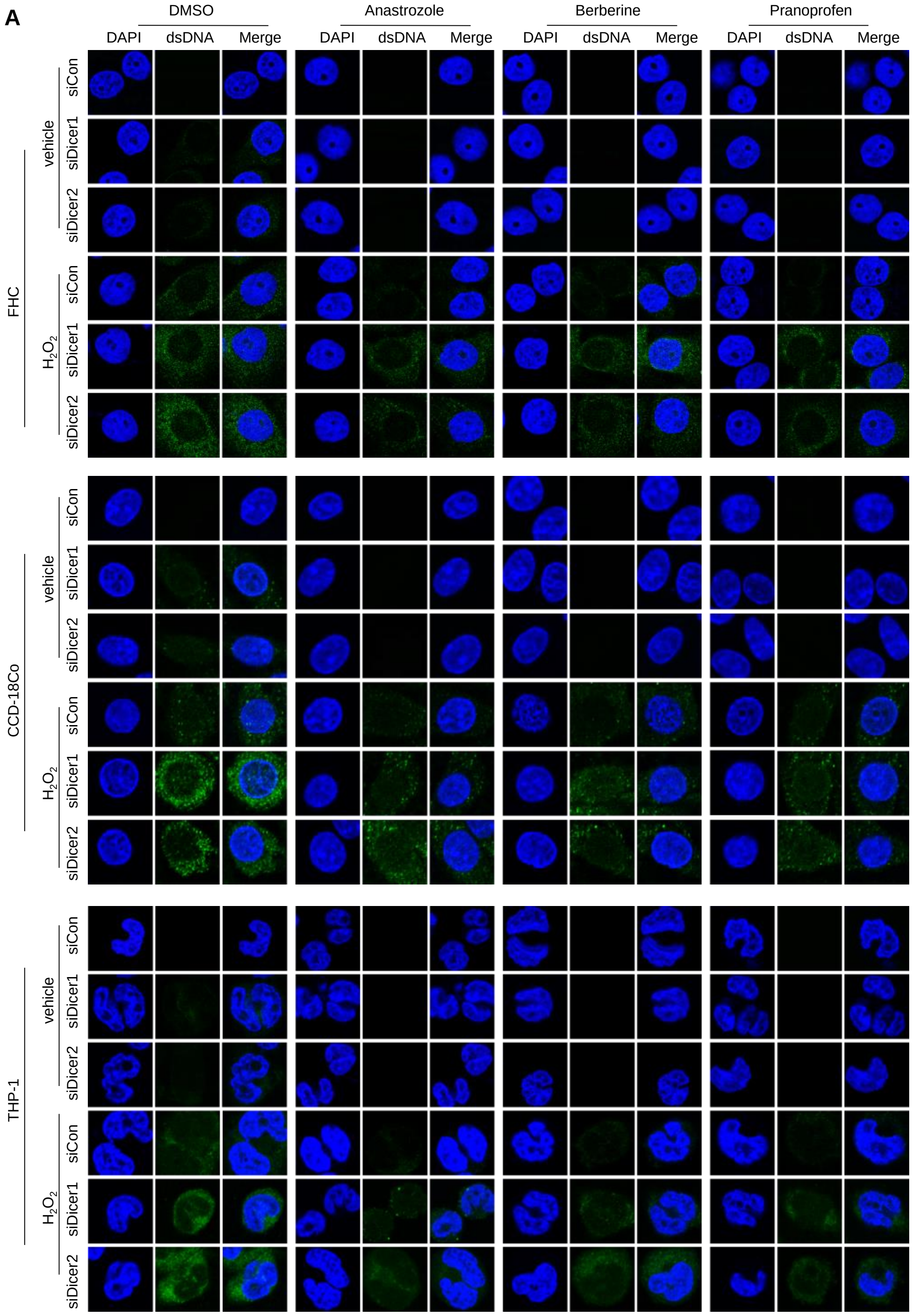
DMSO
Anastrozole
Berberine
Pranoprofen
DAPI
dsDNA
Merge
FHC
CCD-18Co
THP-1
vehicle
H_2O_2
siCon
siDicer1
siDicer2

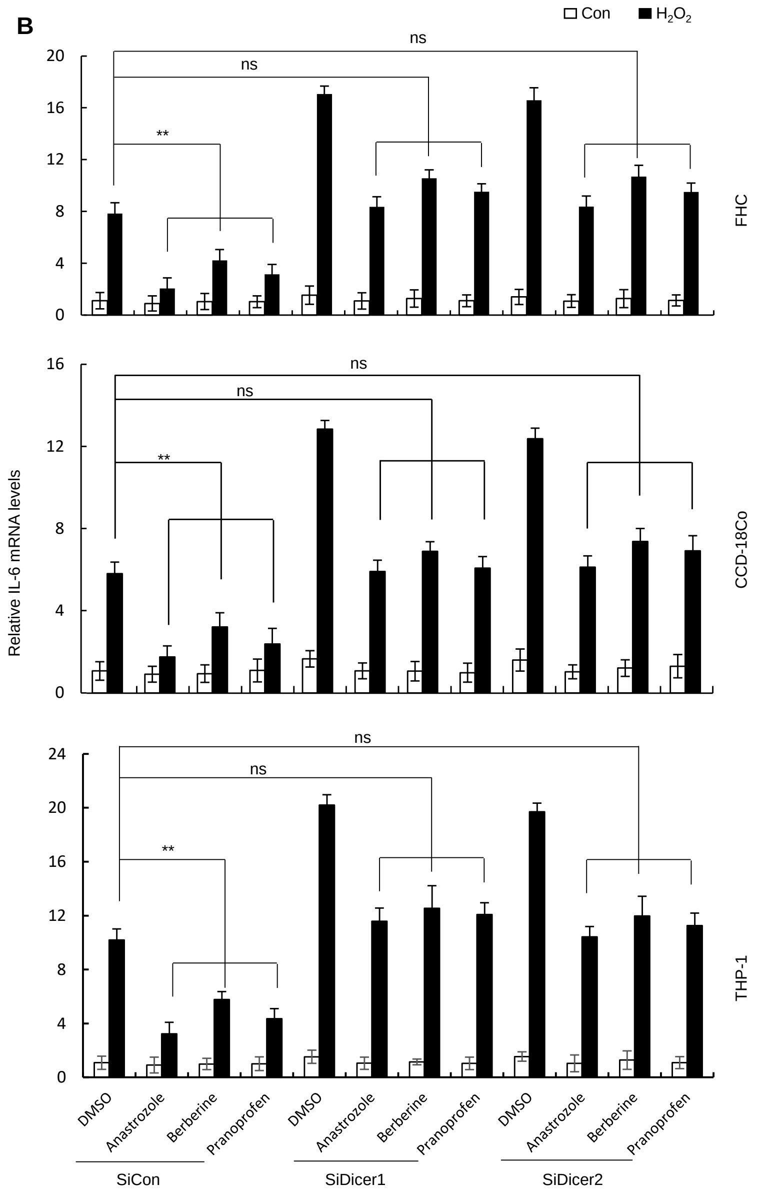

Figure S14. Dicer knockdown partially abrogates the effects of anastrozole, berberine, and pranoprofen on H_2O_2-induced IL-6 expression.

(A, B) FHC, CCD-18Co, and THP-1 cells were transfected with control or Dicer siRNAs, after which 400 µM H_2O_2, 400 µM H_2O_2 + 5 µM anastrozole, 400 µM H_2O_2 + 5 µM berberine, or 400 µM H_2O_2 + 5 µM pranoprofen were added to the culture medium 24 h post-transfection. Cytosolic dsDNA levels (A) and IL-6 mRNA levels (B) were determined 24 h after H_2O_2 treatment. Data represent the means ± SEM. **P < 0.01. ns, not significant.

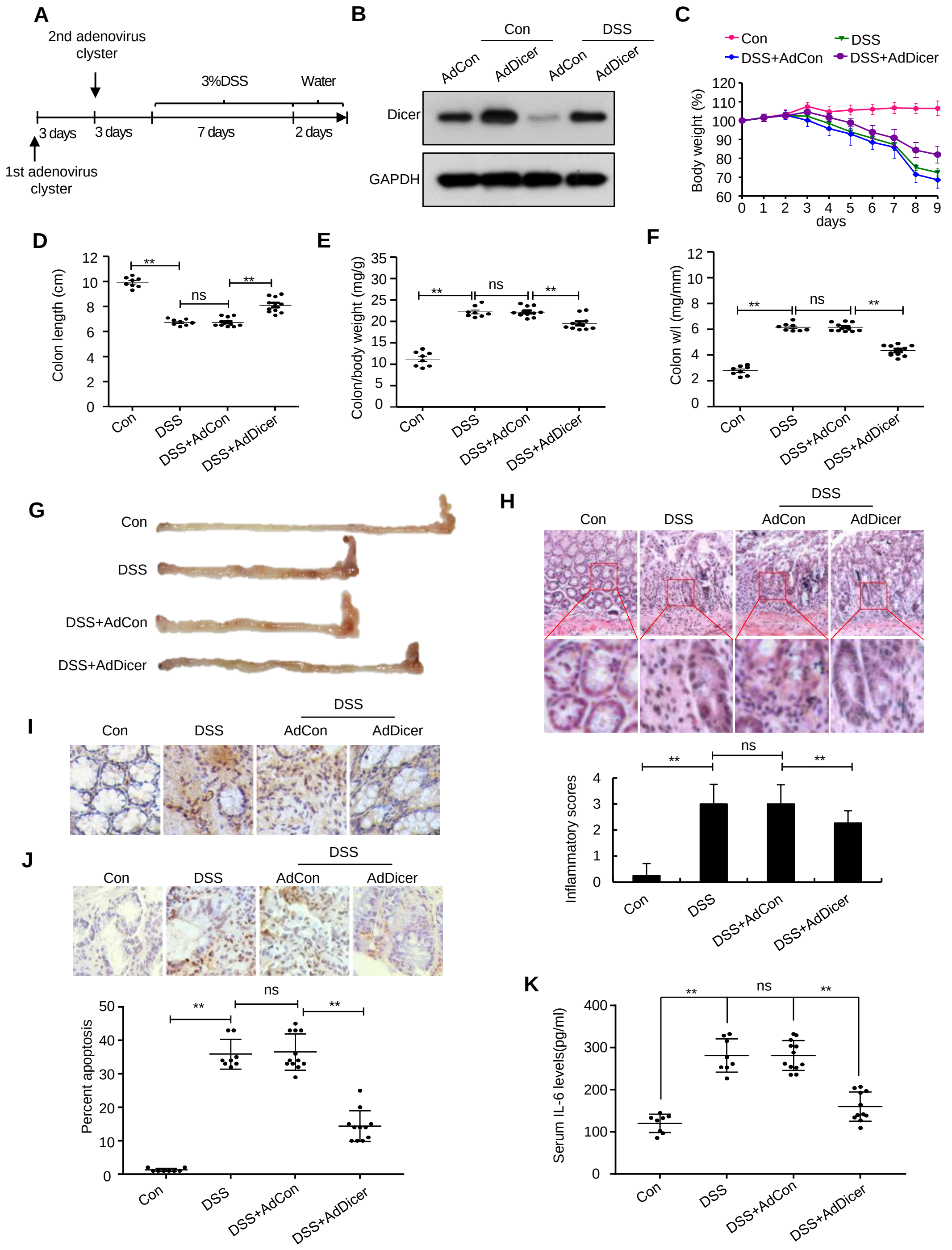

Figure S15. Dicer overexpression alleviates inflammation in colon tissues of the DSS-induced acute colitis mouse model.

(A) Schematic of experimental setup: five-week-old male C57BL/6 mice were intrarectally instilled with adenovirus expressing Dicer or control adenovirus. One week later, acute colitis was induced by oral administration of 3% DSS in drinking water for 7 days followed by 2 days of normal drinking water. Mice that received normal drinking water and were not

instilled with lentivirus were used as control. (B) Dicer expression in colon tissues was determined by western blotting. (C) Relative body weight curves, (D) colon length, (E) colon/body weight ratio, (F) colon weight/length (w/l) ratio, and (G) representative images of mouse gross colon. (H) Representative HE-stained colon sections showing inflammatory infiltrate (upper panel) and inflammatory scores (lower panel). (I) Representative Ly-6G-stained colon sections showing neutrophil infiltrate. (J) Representative images of TUNEL-stained tissue sections (upper panel) and the percentage of apoptotic cells in colon tissues (lower panel). (K) Serum IL-6 levels. Data represent the means ± SEM of at least 8 mice per group. **$P < 0.01$. ns, not significant. DSS: dextran sulfate sodium; HE: hematoxylin and eosin; TUNEL: transferase dUTP nick end labeling.

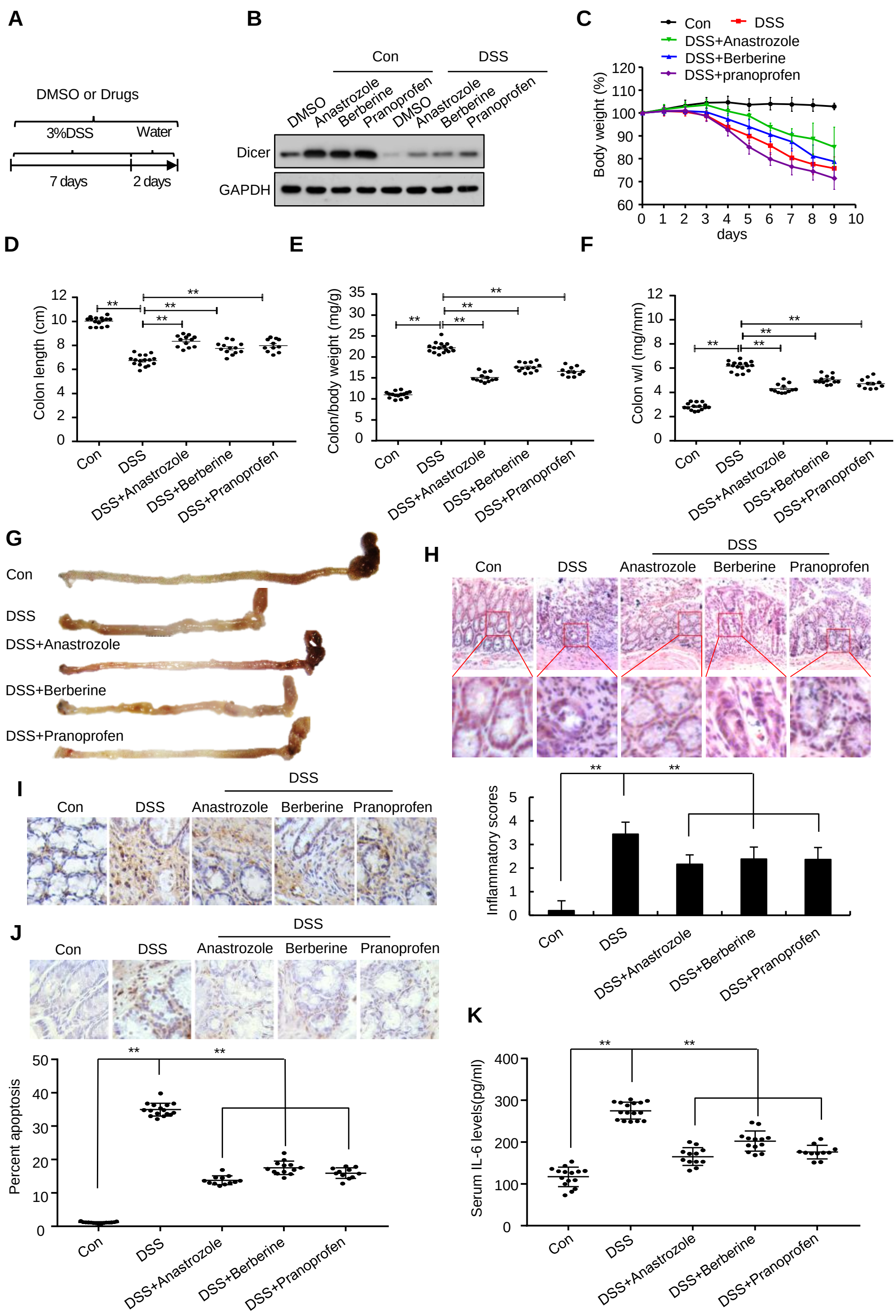

Figure S16. Anastrozole, berberine, and pranoprofen enhance Dicer expression and alleviate inflammation DSS-induced acute colitis.

(A) Schematic of experimental setup: acute colitis was induced in six-week-old male C57BL/6 mice by oral administration of 3% DSS in drinking water for 7 days followed by 2 days of normal drinking water. To rescue Dicer expression in inflamed colon tissues, 20

mg/kg anastrozole, 28 mg/kg berberine, or 16 mg/kg pranoprofen was added to drinking water. All mice were euthanized on day 9. (B) Dicer expression in colon tissues was determined by western blotting. (C) Relative body weight curves, (D) colon length, (E) colon/body weight ratio, (F) colon weight/length (w/l) ratio, and (G) representative images of mouse gross colon. (H) Representative HE-stained colon sections showing inflammatory cell infiltrate (upper panel) and inflammatory scores (lower panel). (I) Representative Ly-6G-stained colon sections showing neutrophil infiltrate. (J) Representative images of TUNEL-stained tissue sections (upper panel) and the percentage of apoptotic cells in colon tissues (lower panel). (K) Serum IL-6 levels. Data represent the means ± SEM of at least 10 mice per group. **P < 0.01. ns, not significant. HE: hematoxylin and eosin; TUNEL: transferase dUTP nick end labeling.

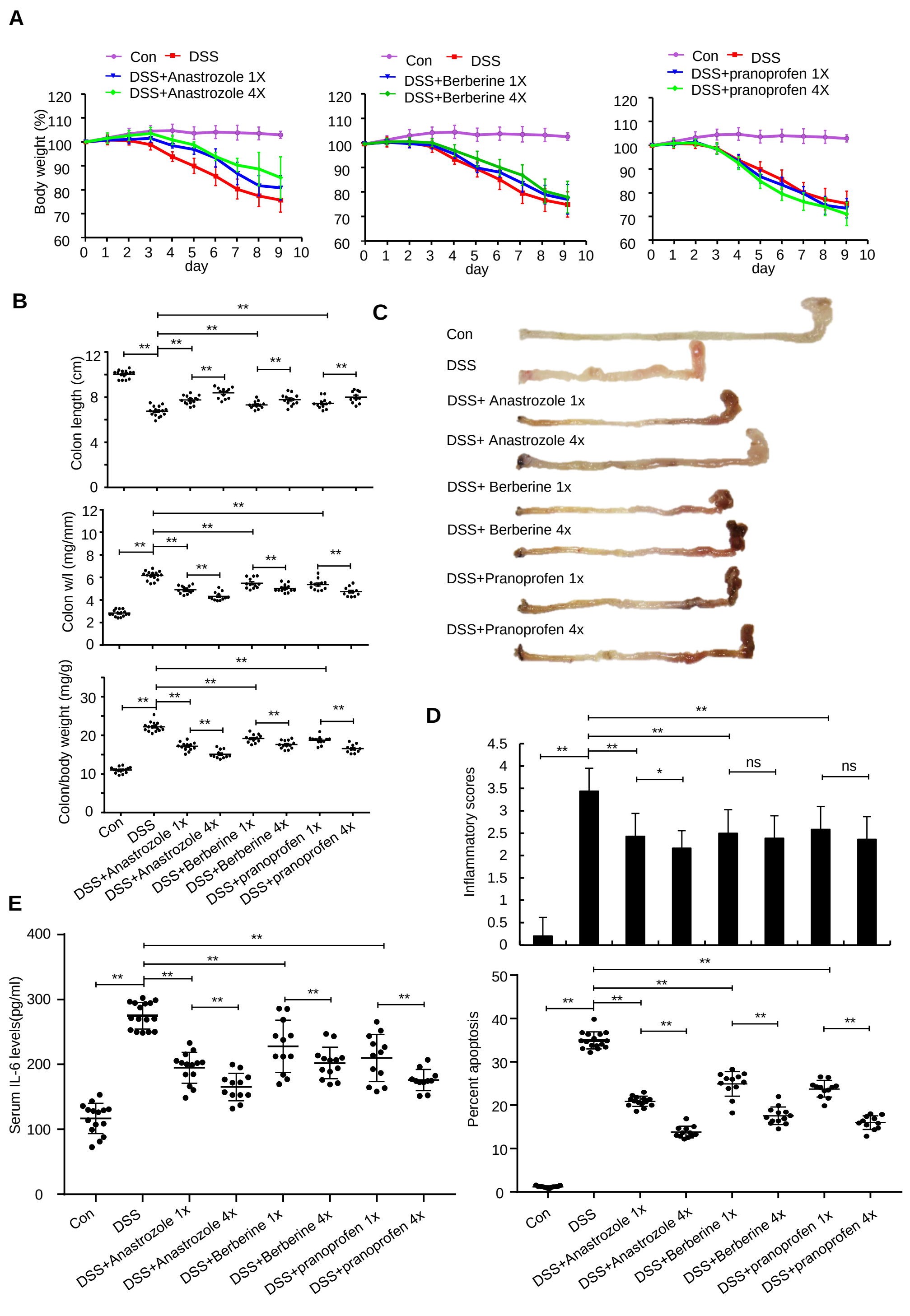

Figure S17. Anastrozole, berberine, and pranoprofen dose-dependently alleviate DSS-induced acute colitis.

Acute colitis was induced in six-week-old male C57BL/6 mice by oral administration of 3% DSS in drinking water for 7 days followed by 2 days of normal drinking water. To rescue Dicer expression in inflamed colon tissues, different doses of anastrozole (1X = 5 mg/kg,

4X = 20 mg/kg), berberine (1X = 7 mg/kg, 4X = 28 mg/kg), or pranoprofen (1X = 4 mg/kg, 4X = 16 mg/kg) were added to drinking water. All mice were euthanized on day 9. (A) Relative body weight curves. (B) Colon length, colon/body weight ratio, and colon weight/length (w/l) ratio. (C) Representative images of mouse gross colon. (D) Inflammatory cell infiltration and percentage of apoptotic cells in colon tissues. (E) Serum IL-6 levels. Data represent the means ± SEM of at least 10 mice per group. *P < 0.05; **P < 0.01. ns, not significant.

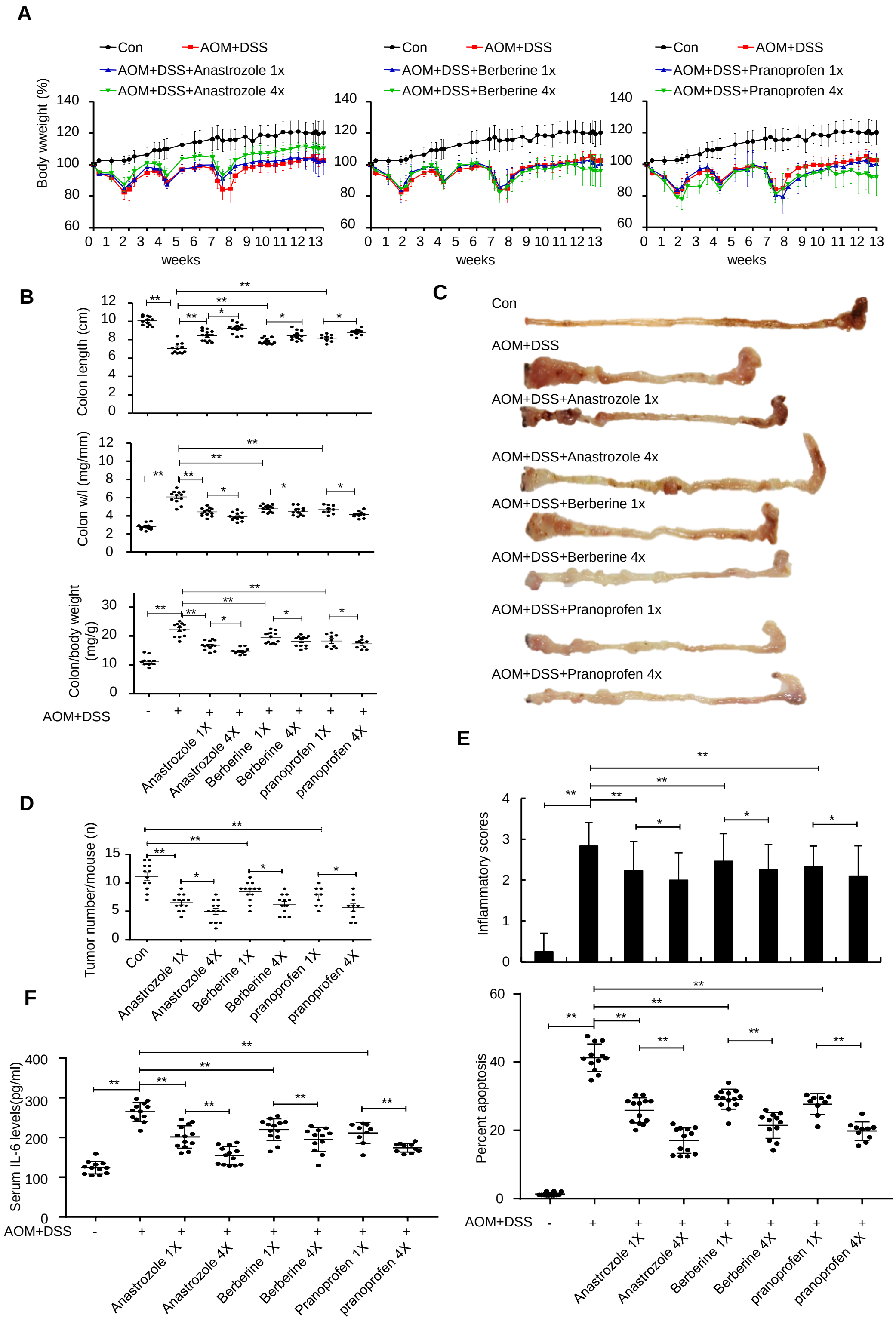

Figure S18. Anastrozole, berberine, and pranoprofen dose-dependently alleviate AOM/DSS-induced chronic colitis and repress colitis-associated carcinogenesis.

Six-week-old male C57BL/6 mice were injected intraperitoneally with 12.5 mg/kg AOM, followed by three cycles of 2.5% DSS treatment. To rescue Dicer expression in inflamed

colon tissues, different doses of anastrozole (1X = 5 mg/kg, 4X = 20 mg/kg), berberine (1X = 7 mg/kg, 4X = 28 mg/kg), or pranoprofen (1X = 4 mg/kg, 4X = 16 mg/kg) were added to drinking water. (A) Relative body weight curves. (B) Colon length, colon/body weight ratio, and colon weight/length (w/l) ratio. (C) Representative images of mouse gross colon. (D) Tumor numbers in the mouse colorectum. (E) Inflammatory cell infiltration and percentage of apoptotic cells in colon tissues. (F) Serum IL-6 levels. Values represent the means ± SEM of at least 10 mice per group. *P < 0.05; **P < 0.01. ns, not significant. AOM: azoxymethane; DSS: dextran sulfate sodium.

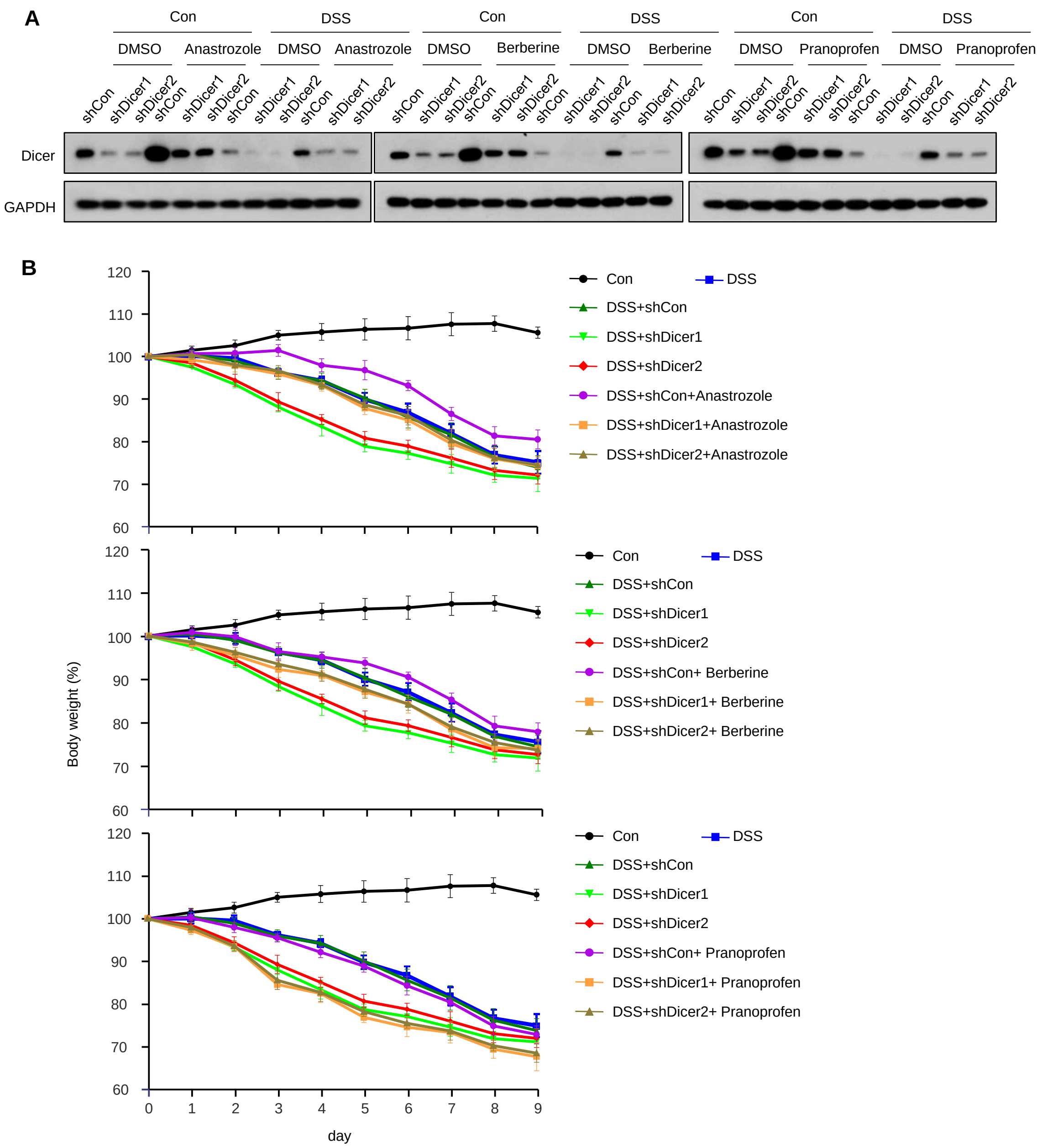
A
Con
DSS
DMSO
Anastrozole
Berberine
Pranoprofen
shCon
shDicer1
shDicer2
Dicer
GAPDH
B
Con
DSS
DSS+shCon
DSS+shDicer1
DSS+shDicer2
DSS+shCon+Anastrozole
DSS+shDicer1+Anastrozole
DSS+shDicer2+Anastrozole
DSS+shCon+ Berberine
DSS+shDicer1+ Berberine
DSS+shDicer2+ Berberine
DSS+shCon+ Pranoprofen
DSS+shDicer1+ Pranoprofen
DSS+shDicer2+ Pranoprofen
Body weight (%)
day

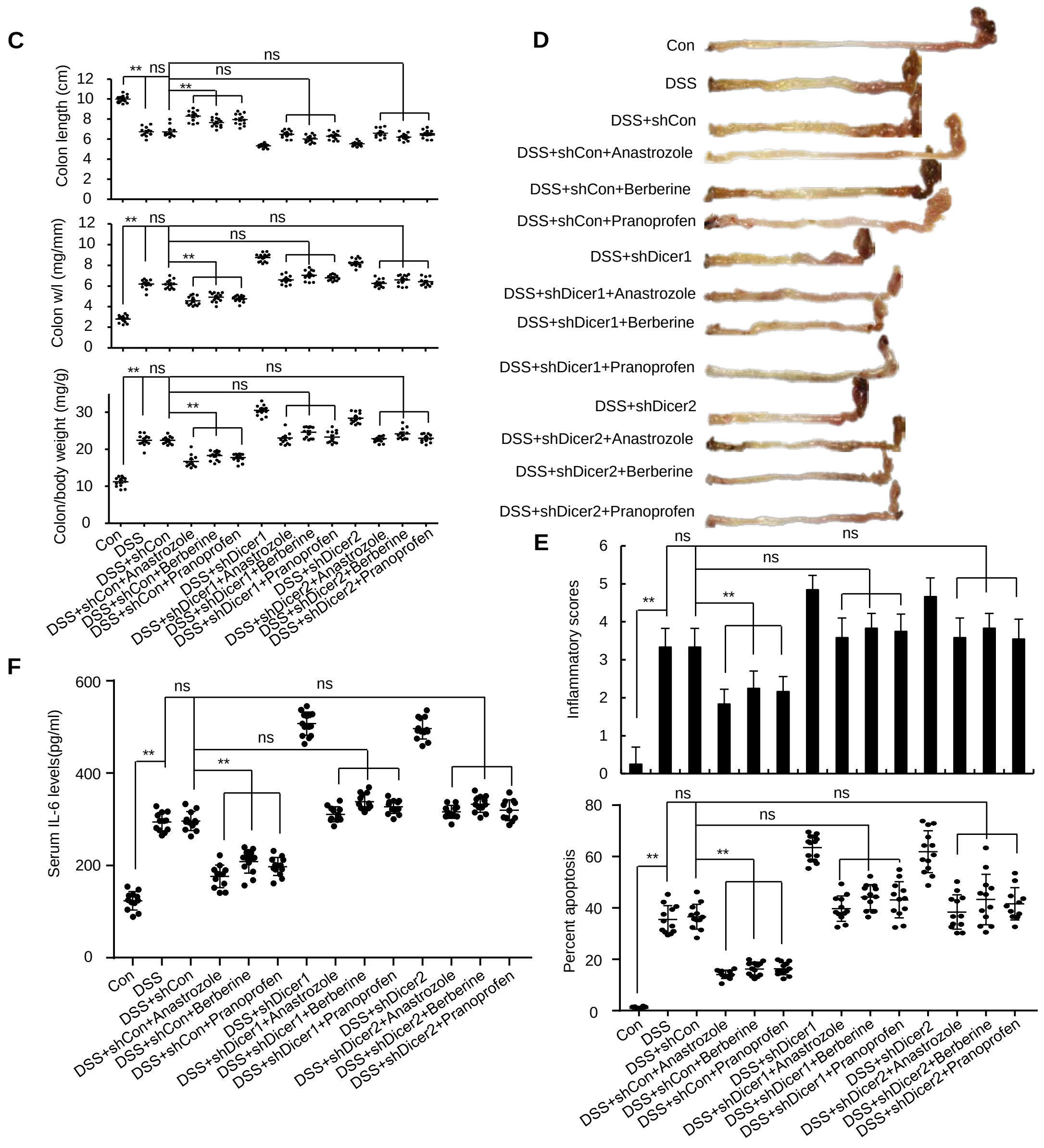

Figure S19. Dicer knockdown partially abrogates the effects of anastrozole, berberine, and pranoprofen on acute inflammation.

Five-week-old male C57BL/6 mice were intrarectally instilled with lentivirus expressing Dicer shRNAs or shCon. One week later, acute colitis was induced by oral administration of 3% DSS in drinking water for 7 days followed by 2 days of normal drinking water. To rescue Dicer expression in inflamed colon tissues, 20 mg/kg anastrozole, 28 mg/kg berberine, or 16 mg/kg pranoprofen was added to drinking water. (A) Dicer expression in colon tissues was determined by western blotting. (B) Relative body weight curves. (C) Colon length, colon/body weight ratio, and colon weight/length (w/l) ratio. (D)

Representative images of mouse gross colon. (E) Inflammatory cell infiltration and percentage of apoptotic cells in colon tissues. (F) Serum IL-6 levels. Data represent the means ± SEM of at least 11 mice per group. **$P < 0.01$. ns, not significant. DSS: dextran sulfate sodium.

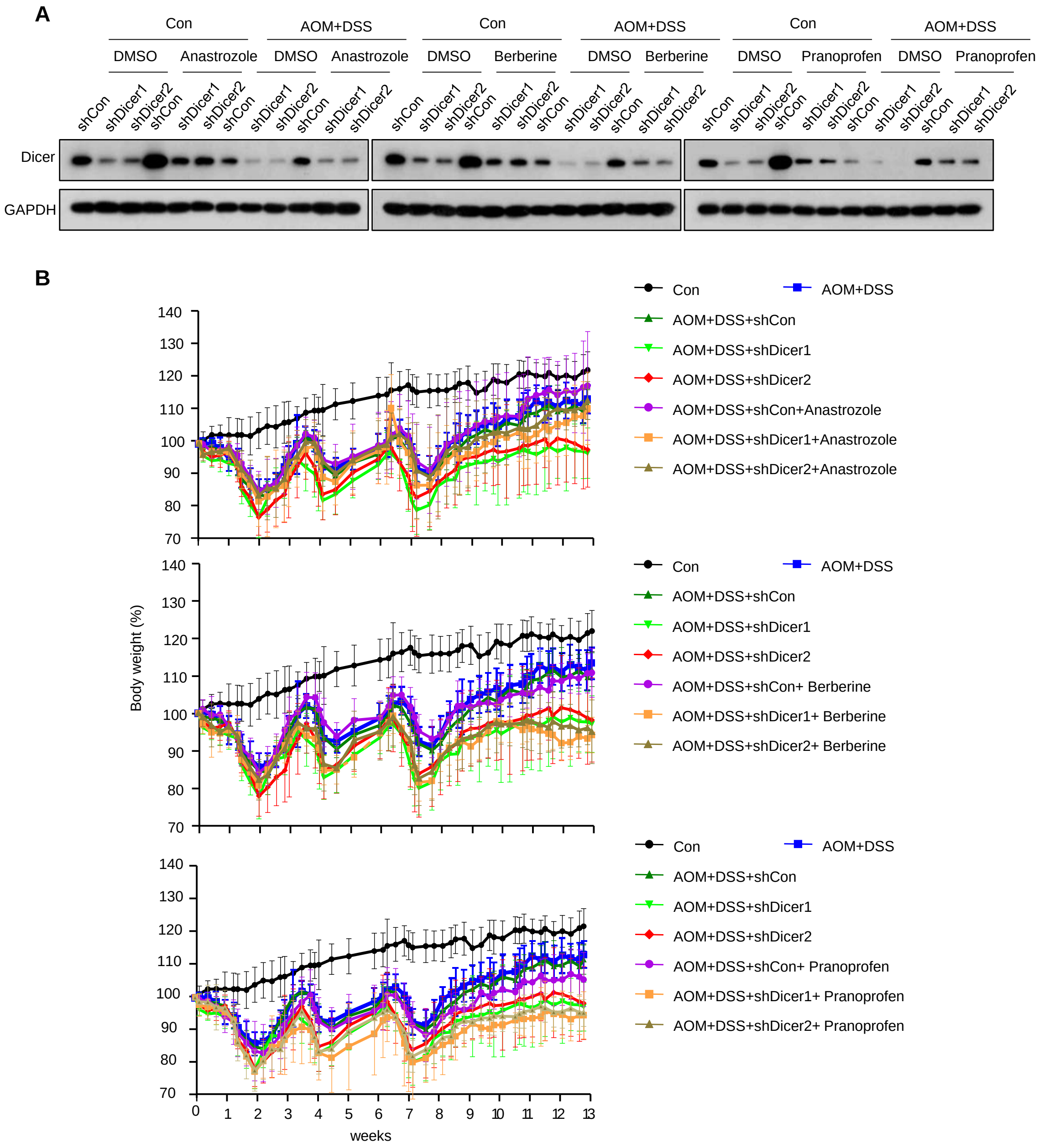
A
Con
AOM+DSS
DMSO
Anastrozole
Berberine
Pranoprofen
shCon
shDicer1
shDicer2
Dicer
GAPDH
B
Con
AOM+DSS
AOM+DSS+shCon
AOM+DSS+shDicer1
AOM+DSS+shDicer2
AOM+DSS+shCon+Anastrozole
AOM+DSS+shDicer1+Anastrozole
AOM+DSS+shDicer2+Anastrozole
AOM+DSS+shCon+ Berberine
AOM+DSS+shDicer1+ Berberine
AOM+DSS+shDicer2+ Berberine
AOM+DSS+shCon+ Pranoprofen
AOM+DSS+shDicer1+ Pranoprofen
AOM+DSS+shDicer2+ Pranoprofen
Body weight (%)
weeks

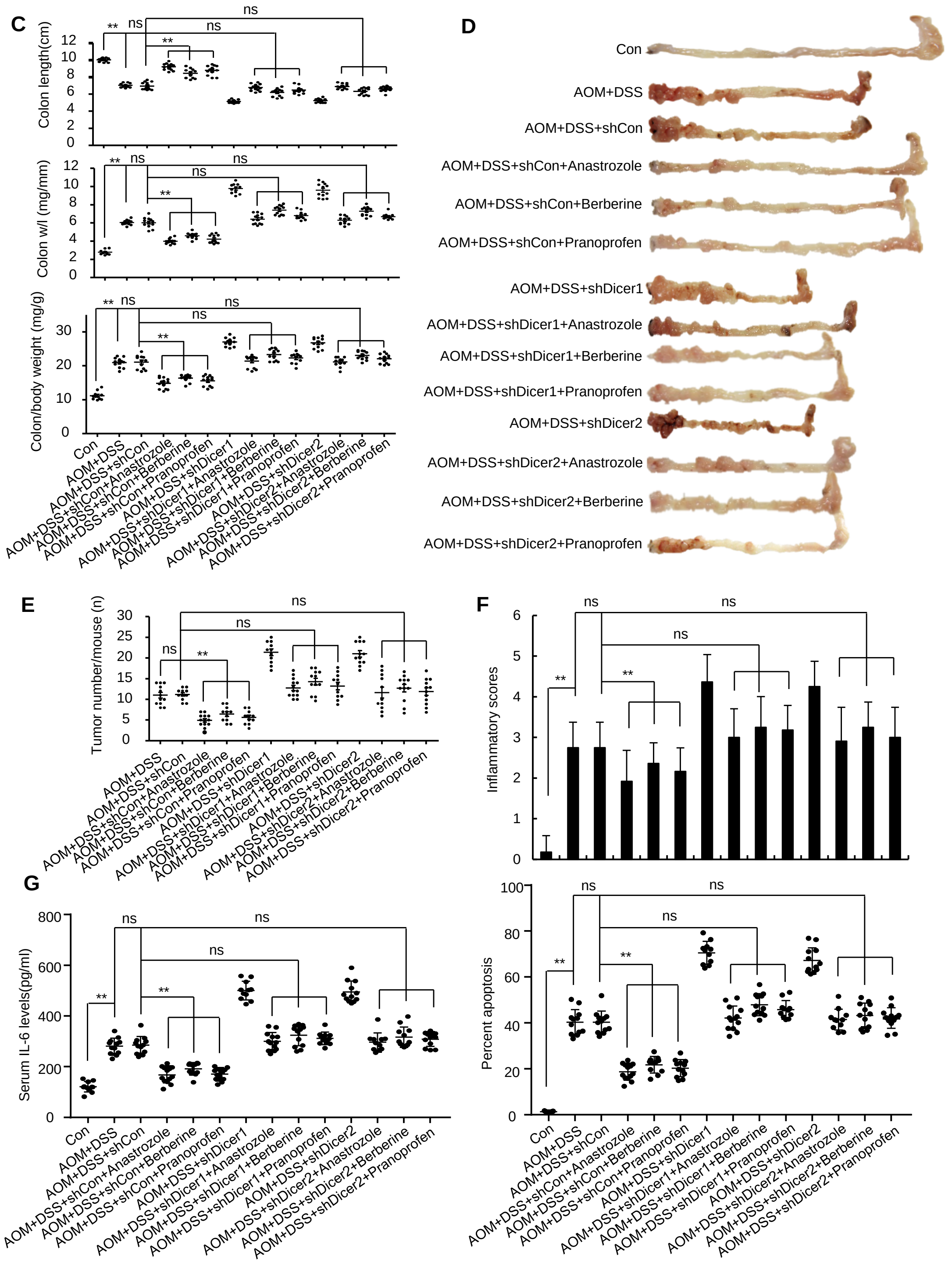

Figure S20. Dicer knockdown partially abrogates the effects of anastrozole, berberine, and pranoprofen on colitis-associated carcinogenesis.

Six-week-old male C57BL/6 mice were injected intraperitoneally with 12.5 mg/kg AOM, followed by three cycles of 2.5% DSS treatment. To knockdown Dicer expression in colon tissues, lentivirus containing the Dicer shRNA expression cassette was intrarectally

administrated to mice three times. To rescue Dicer expression in inflamed colon tissues, 20 mg/kg anastrozole, 28 mg/kg berberine, or 16 mg/kg pranoprofen was added to drinking water. (A) Dicer expression in colon tissues was determined by western blotting. (B) Relative body weight curves. (C) Colon length, colon/body weight ratio, and colon weight/length (w/l) ratio. (D) Representative images of mouse gross colon. (E) Tumor numbers in the mouse colorectum. (F) Inflammatory cell infiltration and percentage of apoptotic cells in colon tissues. (G) Serum IL-6 levels. Data represent the means ± SEM for at least 11 mice per group. **P < 0.01. ns, not significant. AOM: azoxymethane; DSS: dextran sulfate sodium.

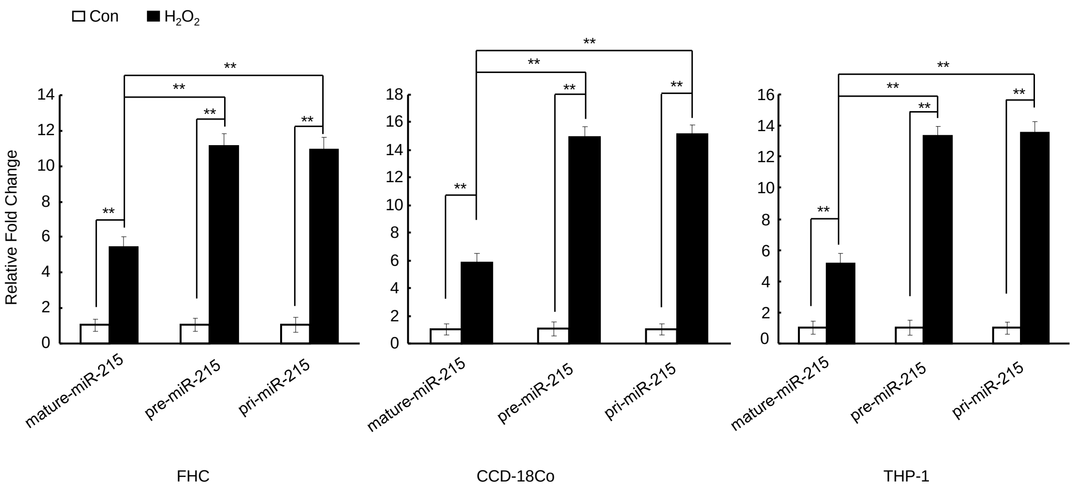

Figure S21. H_2O_2 treatment promotes the expression of pri-miR-215, pre-miR-215, and miR-215.

FHC, CCD-18Co, and THP-1 cells were treated with 400 μM H_2O_2; the levels of pri-miR-215, pre-miR-215, and miR-215 were determined 24 h after treatment. Data represent the means ± SEM of at least 9 mice per group. **P < 0.01.

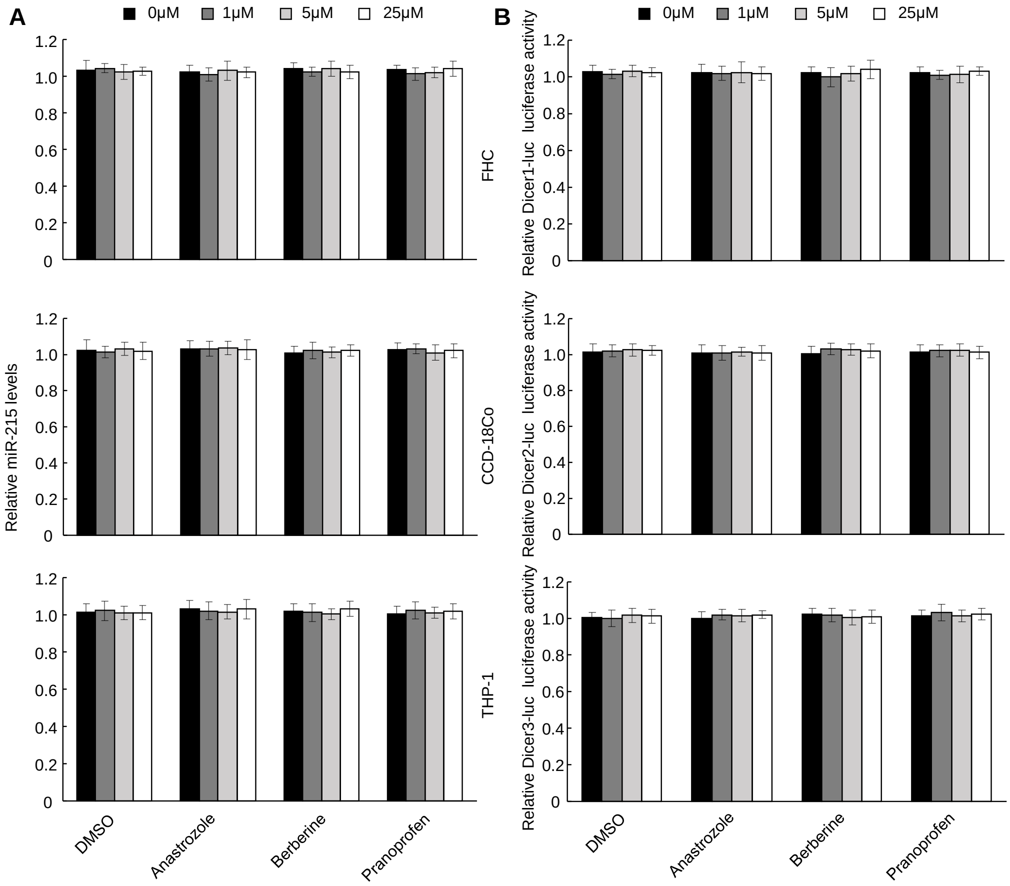

Figure S22. The effects of anastrozole, berberine, and pranoprofen on expression and function of miR-215.

(A) FHC, CCD-18Co, and THP-1 cells were treated with different doses of anastrozole, berberine, and pranoprofen; the levels of miR-215 were determined 24 h after treatment. (B) FHC cells were cotransfected with miR-215 mimics and different reporter plasmids along with a pRL-CMV reporter plasmid, and treated with different doses of anastrozole, berberine, and pranoprofen. Luciferase activity was measured 24 h after drug treatment. Data represent the means ± SEM.

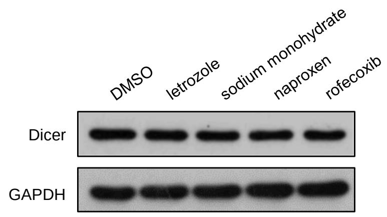

Figure S23. Letrozole, amfenac sodium monohydrate, naproxen, and rofecoxib do not affect Dicer expression.

FHC cells were treated with 30 μM letrozole, amfenac sodium monohydrate, naproxen, or rofecoxib, and Dicer expression was determined by western blotting 24 h after treatment.

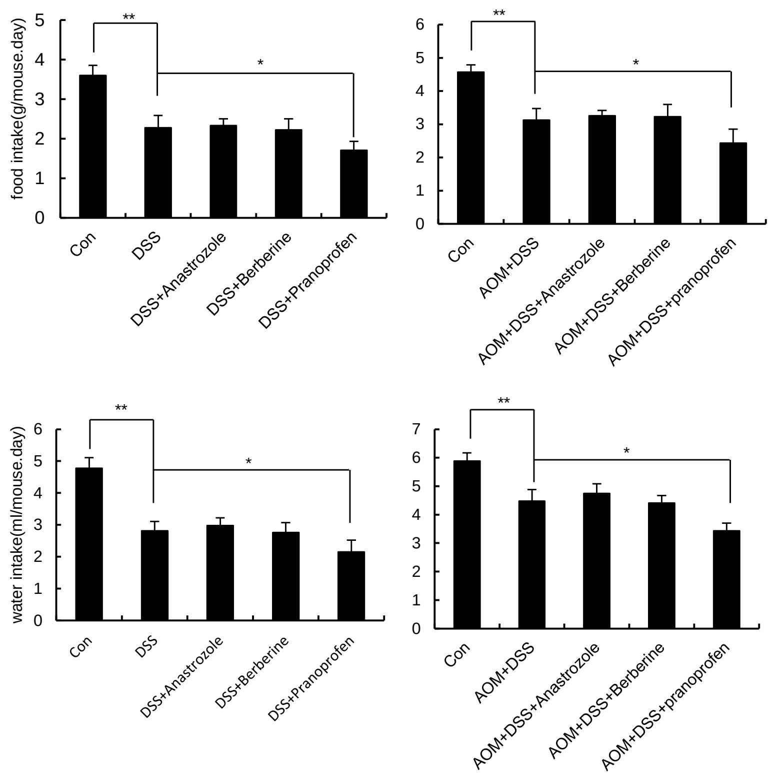

Figure S24. The effects of anastrozole, berberine, and pranoprofen on food and water intake.

Acute or chronic colitis were induced in the presence or absence of anastrozole, berberine, or pranoprofen, the food and water intake of mice was measured. Data represent the means ± SEM of at least 9 mice per group. *P < 0.05; **P < 0.01. ns, not significant.
